# The genetic landscape of ganglioglioma

**DOI:** 10.1186/s40478-018-0551-z

**Published:** 2018-06-07

**Authors:** Melike Pekmezci, Javier E. Villanueva-Meyer, Benjamin Goode, Jessica Van Ziffle, Courtney Onodera, James P. Grenert, Boris C. Bastian, Gabriel Chamyan, Ossama M. Maher, Ziad Khatib, Bette K. Kleinschmidt-DeMasters, David Samuel, Sabine Mueller, Anuradha Banerjee, Jennifer L. Clarke, Tabitha Cooney, Joseph Torkildson, Nalin Gupta, Philip Theodosopoulos, Edward F. Chang, Mitchel Berger, Andrew W. Bollen, Arie Perry, Tarik Tihan, David A. Solomon

**Affiliations:** 1 Department of Pathology, University of California, San Francisco, CA USA; 2 Department of Radiology and Biomedical Imaging, University of California, San Francisco, CA USA; 3 Clinical Cancer Genomics Laboratory, University of California, San Francisco, CA USA; 4 Department of Pathology, Nicklaus Children’s Hospital, Miami, FL USA; 5 Department of Pediatric Hematology/Oncology, Nicklaus Children’s Hospital, Miami, FL USA; 6 Departments of Pathology, Neurology, and Neurosurgery, University of Colorado, Aurora, CO USA; 7 Division of Pediatric Hematology/Oncology, Valley Children’s Hospital, Madera, CA USA; 8 Division of Pediatric Hematology/Oncology, Department of Pediatrics, University of California, San Francisco, CA USA; 9 Department of Neurological Surgery, University of California, San Francisco, CA USA; 10 Department of Neurology, University of California, San Francisco, CA USA; 11 Division of Neuro-Oncology, Department of Neurological Surgery, University of California, San Francisco, CA USA; 12 Division of Pediatric Hematology/Oncology, UCSF Benioff Children’s Hospital Oakland, Oakland, CA USA

**Keywords:** Ganglioglioma, Epilepsy, Seizures, Glioneuronal tumor, Targeted next-generation sequencing, Ras-Raf-MEK-ERK, MAP kinase signaling pathway, *BRAF*, *KRAS*, *RAF1*, *NF1*, *FGFR1*, *FGFR2*, *ABL2*

## Abstract

Ganglioglioma is the most common epilepsy-associated neoplasm that accounts for approximately 2% of all primary brain tumors. While a subset of gangliogliomas are known to harbor the activating p.V600E mutation in the *BRAF* oncogene, the genetic alterations responsible for the remainder are largely unknown, as is the spectrum of any additional cooperating gene mutations or copy number alterations. We performed targeted next-generation sequencing that provides comprehensive assessment of mutations, gene fusions, and copy number alterations on a cohort of 40 gangliogliomas. Thirty-six harbored mutations predicted to activate the MAP kinase signaling pathway, including 18 with *BRAF* p.V600E mutation, 5 with variant *BRAF* mutation (including 4 cases with novel in-frame insertions at p.R506 in the β3-αC loop of the kinase domain), 4 with *BRAF* fusion, 2 with *KRAS* mutation, 1 with *RAF1* fusion, 1 with biallelic *NF1* mutation, and 5 with *FGFR1/2* alterations. Three gangliogliomas with *BRAF* p.V600E mutation had concurrent *CDKN2A* homozygous deletion and one additionally harbored a subclonal mutation in *PTEN*. Otherwise, no additional pathogenic mutations, fusions, amplifications, or deletions were identified in any of the other tumors. Amongst the 4 gangliogliomas without canonical MAP kinase pathway alterations identified, one epilepsy-associated tumor in the temporal lobe of a young child was found to harbor a novel *ABL2*-*GAB2* gene fusion. The underlying genetic alterations did not show significant association with patient age or disease progression/recurrence in this cohort. Together, this study highlights that ganglioglioma is characterized by genetic alterations that activate the MAP kinase pathway, with only a small subset of cases that harbor additional pathogenic alterations such as *CDKN2A* deletion.

**Electronic supplementary material:**

The online version of this article (10.1186/s40478-018-0551-z) contains supplementary material, which is available to authorized users.

## Introduction

Ganglioglioma is a well-differentiated and typically slow-growing glioneuronal neoplasm composed of dysplastic ganglion cells in combination with neoplastic glial cells [2]. They often arise in the temporal lobe of children and young adults in association with seizures. However, they can occur at any age and throughout the neuraxis including the cerebellum, brainstem, and spinal cord. The neuroimaging appearance is variable, but they often display a mix of solid and cystic components. Most gangliogliomas correspond histologically to WHO grade I and do not recur after complete resection. However, gangliogliomas are both histologically and clinically variable, and tumor recurrence or anaplastic progression occurs in a subset of cases.

The activating p.V600E hotspot mutation in the *BRAF* oncogene has been identified in a subset of gangliogliomas, ranging from approximately 10–60% depending on the study and anatomic site, with highest frequencies reported in cortical tumors and lower frequency reported in spinal cord tumors [6, 7, 9, 11–13, 16, 21, 27, 30, 31, 36–38]. However, *BRAF* p.V600E mutation is not specific to ganglioglioma and has been described in a wide spectrum of neuroepithelial tumors including pilocytic astrocytoma, dysembryoplastic neuroepithelial tumor (DNET), pediatric IDH-wildtype diffuse astrocytoma, polymorphous low-grade neuroepithelial tumor of the young (PLNTY), pleomorphic xanthoastrocytoma, and epithelioid glioblastoma [6, 12, 17, 22, 30, 31, 36, 38]. Additionally, the genetic alterations responsible for *BRAF* p.V600 wildtype gangliogliomas are largely unknown, as is the spectrum of any additional cooperating gene mutations or copy number alterations. Herein, we performed comprehensive molecular profiling on a cohort of 40 pathologically-confirmed gangliogliomas in order to evaluate the genetic landscape of this tumor entity and identify any genetic alterations that may correlate with differences in clinical outcomes or imaging and histologic features.

## Methods

### Patients and tumor tissue

We searched our institutional pathology archives for cases with a diagnosis of ganglioglioma, spanning years 1990 to 2017. Cases with available diagnostic slides and tissue blocks containing sufficient tumor tissue for genetic analysis were included. All tumor specimens had been fixed in 10% neutral-buffered formalin and embedded in paraffin. Pathologic review of all tumor samples was performed to confirm the diagnosis by a group of five expert neuropathologists (M.P., A.W.B., A.P., T.T., and D.A.S.) with a unanimous consensus diagnosis established for all included cases. All tumors contained an unequivocal ganglion cell component admixed with a neoplastic glial component. Tumors that were better classified as other diagnostic entities (e.g. pleomorphic xanthoastrocytoma, pilocytic astrocytoma, DNET, PLNTY, multinodular and vacuolating neuronal tumor of the cerebrum [MVNT], and low-grade glial/glioneuronal neoplasm not further classifiable) were excluded. Histologic features including morphology of the glial component and presence of eosinophilic granular bodies, Rosenthal fibers, calcifications, myxoid background, CD34-immunopositive ramified cells, perivascular lymphocytes, mitotic activity, necrosis, microvascular proliferation, and leptomeningeal spread were assessed. Pre-operative imaging was reviewed for all available cases (*n* = 29) by an expert neuroradiologist (J.E.V.). Imaging features assessed were tumor location, size, circumscription, cortical involvement, subcortical white matter involvement, multinodularity, cystic component, T1 intensity, T2 intensity, contrast enhancement, calcifications, hemorrhage, and overlying bony remodeling. Clinical data was extracted from institutional electronic medical records including patient age, sex, presenting symptomatology, duration of symptoms, extent of surgery, adjuvant therapy, and follow-up interval. Event-free survival was defined as time until recurrence after gross total resection or disease progression after subtotal resection based on either imaging impression or pathologic confirmation.

### Genomic DNA extraction and targeted next-generation sequencing

Genomic DNA was extracted from tumor tissue that had been macrodissected from formalin-fixed, paraffin-embedded blocks or unstained sections using the QIAamp DNA FFPE Tissue Kit (Qiagen) according to the manufacturer’s protocol. Tumor tissue from the initial resection was used in 35 patients, and tumor tissue from a second surgery after recurrence/progression was used in 5 patients (SF-GG-3, SF-GG-5, SF-GG-18, SF-GG-23, and SF-GG-35). Capture-based next-generation DNA sequencing was performed as previously described at the UCSF Clinical Cancer Genomics Laboratory, using an assay that targets all coding exons of 479 cancer-related genes, *TERT* promoter, select introns and upstream regulatory regions of 47 genes to enable detection of structural variants including gene fusions, and DNA segments at regular intervals along each chromosome to enable genome-wide copy number and zygosity analysis, with a total sequencing footprint of 2.8 Mb (UCSF500 Cancer Panel; Additional file 1: Table S1) [20]. Sequencing libraries were prepared from genomic DNA, and target enrichment was performed by hybrid capture using a custom oligonucleotide library (Roche NimbleGen). Sequencing was performed on an Illumina HiSeq 2500. Duplicate sequencing reads were removed computationally to allow for accurate allele frequency determination and copy number calling. The analysis was based on the human reference sequence (NCBI build 37) using the following software packages: BWA, Samtools, Picard tools, GATK, CNVkit, Pindel, SATK, Annovar, Freebayes, and Delly. Single nucleotide variants, insertions/deletions, and structural variants were visualized and verified using the Integrated Genome Viewer. Genome-wide copy number analysis based on on-target and off-target reads was performed by CNVkit and Nexus Copy Number (Biodiscovery). As the majority of cases were analyzed as tumor-only without a paired normal sample to accurately confirm the somatic status of variants, only those variants classified as pathogenic or likely pathogenic are reported herein. Variants of unknown significance are not reported, given that the vast majority of these likely represent rare or private germline variants and not somatic mutations.

### Statistical analysis

Statistical analysis was performed using GraphPad Prism software version 7. Kaplan-Meier event-free survival analysis for patients with ganglioglioma stratified by molecular alterations was performed using Log-rank (Mantel-Cox) test. Comparison of clinical, imaging, and histologic features stratified by molecular alterations was performed using Fisher’s exact test.

## Results

### Demographic and clinical features of the ganglioglioma cohort

Forty patients with pathologically confirmed ganglioglioma were included in this study (Table 1 and Additional file 1: Table S2). The 23 male and 17 female patients ranged from 0 to 64 years of age (median 21 years). The presenting symptoms were variable and ranged from seizures in patients with temporal lobe tumors to extremity weakness in patients with spinal cord tumors. Thirty-one tumors (78%) were located in the cerebral hemispheres with 19 in the temporal lobe, three in the frontal lobe, four in the parietal lobe, and five in the occipital lobe. Four tumors were located in the cerebellum, two were located in the thalamus, and three were located in the spinal cord (Additional file 1: Table S3). The available clinical follow-up after initial surgical intervention ranged from 0 to 29 years (median 1.8 years). Gross total resection was achieved in 26 patients, two of which had subsequent tumor recurrence at 1.2 and 7.8 years. Subtotal resection was performed in 11 patients, four of which showed subsequent tumor progression (0.6 to 10 years later). Extent of resection was unknown in three patients, two of which had subsequent tumor progression at 1.4 and 1.8 years.

**Table 1:** Summary of the clinicopathologic features and molecular alterations in the ganglioglioma patient cohort

Tumor ID	Age/sex	Tumor location	Presenting symptoms	Radiographic pattern	Glial component	Pathogenic genetic alterations identified	Chromosomal gains/losses	Extent of resection	Recurrence or progression	Time to recurrence or progression (years)	Length of follow-up (years)
SF-GG-01	5 M	Temporal lobe	seizures	cystic and solid	astrocytic	BRAF p.V600E	none	gross total	no	0.1	0.1
SF-GG-02	8 M	Occipital lobe	seizures	multicystic	astrocytic	BRAF p.V600E	none	gross total	no	0.4	0.4
SF-GG-03	14 M	Occipital lobe	seizures	N/A	astrocytic	BRAF p.V600E, CDKN2A/B HD, PTEN p.R173C (s)	+distal 3q, − 9	gross total	yes (P)	1.8	1.8
SF-GG-04	11 M	Temporal lobe	seizures	N/A	astrocytic	BRAF p.V600E	none	subtotal	yes (R)	4.4	4.4
SF-GG-05	12 F	Temporal lobe	seizures	N/A	astrocytic	BRAF p.V600E	+ 7, + 9, + 12	unknown	yes (P)	1.8	1.8
SF-GG-06	12 F	Temporal lobe	seizures	cystic and solid	astrocytic	BRAF p.V600E	none	gross total	no	0.5	0.5
SF-GG-07	30 M	Temporal lobe	seizures	N/A	astrocytic	BRAF p.V600E	-1p, −16q	gross total	no	0.0	0.0
SF-GG-08	27 M	Temporal lobe	seizures	N/A	astrocytic	BRAF p.V600E	+ 5, + 7, + 8, + 11, + 12, + 15, + 16, + 19, + 20, + 21	gross total	no	0.1	0.1
SF-GG-09	34 M	Temporal lobe	seizures	solid	astrocytic	BRAF p.V600E, CDKN2A/B HD	-9	gross total	no	0.4	0.4
SF-GG-10	37 M	Temporal lobe	seizures	N/A	astrocytic	BRAF p.V600E	+ 5	gross total	no	9.4	9.4
SF-GG-11	25 F	Temporal lobe	seizures	cyst with mural nodule	astrocytic	BRAF p.V600E, CDKN2A/B HD	-1p, −9	gross total	no	6.7	6.7
SF-GG-12	41 F	Temporal lobe	seizures	cystic and solid	astrocytic	BRAF p.V600E	none	gross total	no	9.2	9.2
SF-GG-13	63 M	Parietal lobe	seizures	cyst with mural nodule	astrocytic	BRAF p.V600E	-1p, +19p	gross total	no	0.0	0.0
SF-GG-14	15 F	Cerebellum	headaches	cystic and solid	astrocytic	BRAF p.V600E	none	gross total	no	14.8	14.8
SF-GG-15	13 M	Cerebellum	asymptomatic	complex heterogeneous	astrocytic	BRAF p.V600E	none	subtotal	yes (P)	10.4	10.4
SF-GG-16	30 F	Cerebellum	nystagmus	complex heterogeneous	astrocytic	BRAF p.V600E	none	subtotal	no	13.8	13.8
SF-GG-17	8 M	Thalamus	headache	complex heterogeneous	astrocytic	BRAF p.V600E	none	biopsy	no	0.0	0.0
SF-GG-18	12 M	Thalamus	dystonia	N/A	astrocytic	BRAF p.V600E	none	biopsy	yes (P)	29.3	29.3
SF-GG-19	7 M	Occipital lobe	seizures	cyst with mural nodule	astrocytic	BRAF p.T599_W604delinsTDG	none	gross total	no	14.1	14.1
SF-GG-20	19 F	Temporal lobe	seizures	solid	astrocytic	BRAF p.R506delinsRVLR	none	gross total	no	0.2	0.2
SF-GG-21	29 M	Temporal lobe	seizures	cystic and solid	astrocytic	BRAF p.R506delinsRSTQ	+ 5, + 6, + 7, + 11, + 18	subtotal	no	1.8	1.8
SF-GG-22	23 F	Temporal lobe	seizures	cyst with mural nodule	astrocytic	BRAF p.L505delinsLEYLS	none	unknown	no	0.0	0.0
SF-GG-23	41 F	Parietal lobe	visual distortion	solid multinodular	astrocytic	BRAF p.R506delinsRVLR	none	gross total	yes (P)	8.1	8.1
SF-GG-24	5 F	Spinal cord	asymptomatic	cystic and solid	astrocytic	KIAA1549-BRAF fusion	none	subtotal	no	0.2	0.2
SF-GG-25	17 F	Spinal cord	leg weakness	complex heterogeneous	astrocytic	KIAA1549-BRAF fusion	none	gross total	no	4.5	4.5
SF-GG-26	9 F	Temporal lobe	seizures	complex heterogeneous	astrocytic	CDC42BPB-BRAF fusion	-interstitial 17p	subtotal	yes (R)	2.2	2.2
SF-GG-27	8 F	Occipital lobe	seizures	solid	astrocytic	KLHL7-BRAF fusion	+ 7	gross total	no	1.4	1.4
SF-GG-28	48 M	Frontal lobe	seizures	cyst with mural nodule	astrocytic	ERC2-RAF1 fusion	+ 3, −interstitial 3p, + 5, + 7	gross total	no	0.5	0.5
SF-GG-29	32 F	Insula	seizures	cyst with mural nodule	astrocytic	KRAS p.Q61K	none	gross total	no	0.0	0.0
SF-GG-30	24 M	Temporal lobe	seizures	cyst with mural nodule	astrocytic	KRAS p.Q61K	none	gross total	no	1.0	1.0
SF-GG-31	28 M	Temporal lobe	headaches	cystic and solid	astrocytic	germline NF1 p.Q514frameshift w/ somatic LOH	+ 4, + 5, + 7, + 8, + 9, + 12, + 15, −17p, −18p, + 22	gross total	no	5.9	5.9
SF-GG-32	35 M	Frontal lobe	paresthesias	solid	oligodendroglial	FGFR1-TACC1 fusion	none	subtotal	no	1.4	1.4
SF-GG-33	59 F	Temporal lobe	seizures	multicystic	astrocytic	FGFR1 p.N546 K	none	gross total	no	5.9	5.9
SF-GG-34	7 M	Temporal lobe	seizures	N/A	oligodendroglial	FGFR2-KIAA1598 fusion	none	gross total	no	5.3	5.3
SF-GG-35	35 M	Parietal lobe	unknown	complex heterogeneous	oligodendroglial	FGFR2-INA fusion	-portions of 10	unknown	yes (P)	9.1	9.1
SF-GG-36	37 F	Parietal lobe	leg weakness	N/A	astrocytic	FGFR2 exon 17 splice site mutation	none	subtotal	no	7.5	7.5
SF-GG-37	2 M	Frontal lobe	seizures	microcystic	astrocytic	ABL2-GAB2 fusion	none	subtotal	no	0.3	0.3
SF-GG-38	53 F	Occipital lobe	asymptomatic	N/A	astrocytic	none identified	none	gross total	no	0.0	0.0
SF-GG-39	0 F	Cerebellum	nystagmus	cystic and solid	astrocytic	none identified	none	gross total	no	14.9	14.9
SF-GG-40	10 M	Spinal cord	arm weakness	N/A	astrocytic	none identified	none	gross total	no	14.3	14.3

*HD* Homozygous deletion. *LOH* Loss of heterozygosity. (s), subclonal. (R), radiographic recurrence/progression. (P), pathologically confirmed recurrence/progression

### Histopathologic features of the ganglioglioma cohort

All 40 gangliogliomas contained dysmorphic ganglion cells admixed with a neoplastic glial component (Additional file 1: Table 4 and Additional file 2: Figure S1). The glial component demonstrated astrocytic morphology in 37 cases (93%) and oligodendroglial morphology in three cases (8%). Eosinophilic granular bodies were present in 27 cases (68%), and Rosenthal fibers were present in six cases (15%). Calcifications were present in 19 cases (48%), and were extensive in eight of these. CD34 immunopositive ramified cells were present in 16 of 19 evaluated cases (84%). None of the tumors harbored anaplastic features, such as high mitotic index (more than 2 per 10 high power fields), necrosis, or microvascular proliferation.

### Genetic alterations identified in the ganglioglioma cohort

**Fig. 1:**
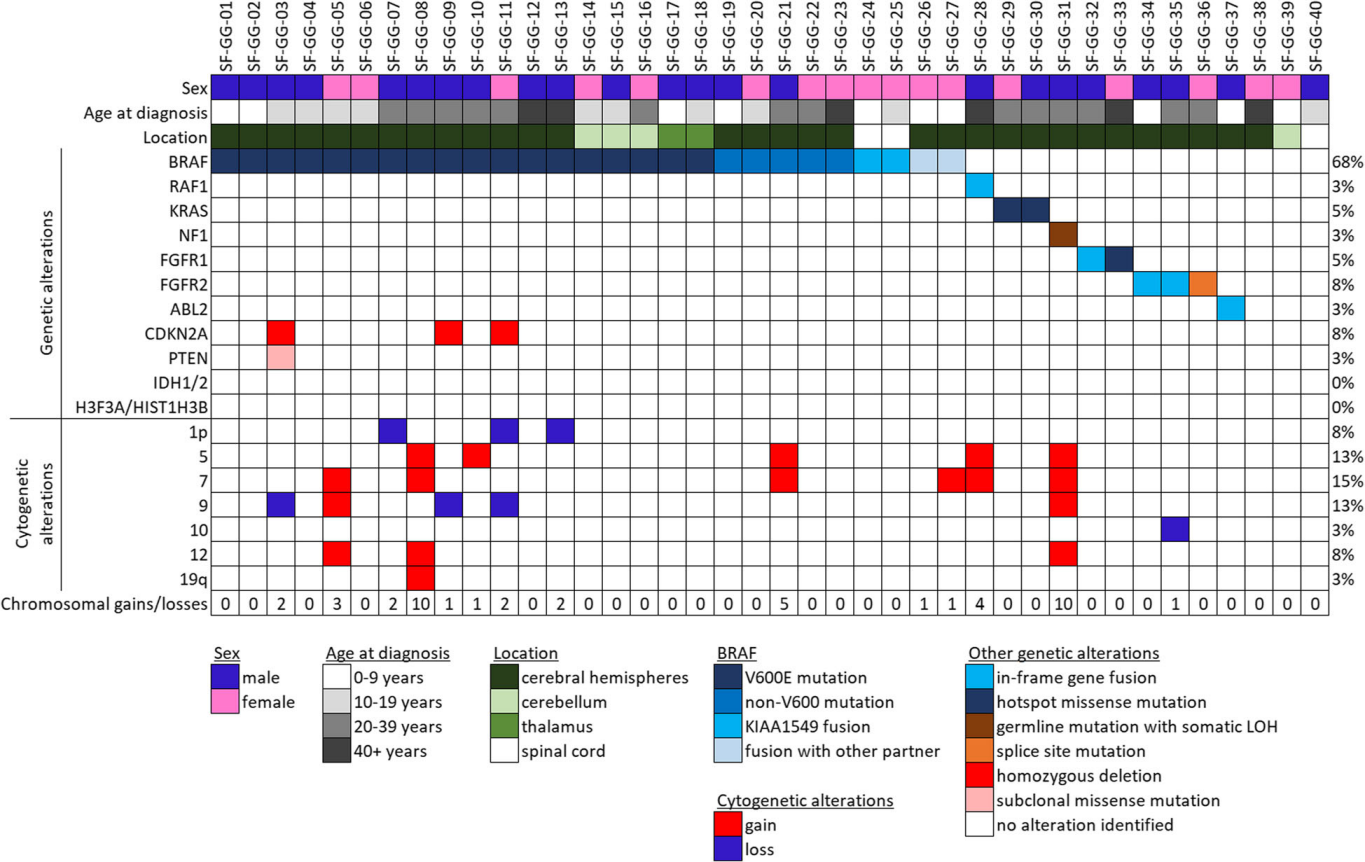
Oncoprint summary table of the 40 patients with ganglioglioma including patient age, sex, tumor location, genetic alterations, and number of chromosomal gains/losses

Targeted next-generation sequencing that provides assessment of mutations, gene fusions, amplifications, deletions, and chromosomal copy number alterations was performed on the cohort of 40 gangliogliomas (Fig. 1 and Additional file 1: Tables S5–S7). Twenty-seven of the tumors harbored pathogenic alterations in the *BRAF* oncogene, including 18 with p.V600E hotspot mutation, five with non-V600E variant mutations (p.L505delinsLEYLS, p.R506delinsRVLR [in two cases], p.R506delinsRSTQ, and p.T599_W604delinsTDG), and four with in-frame gene fusions (two with *KIAA1549* as the fusion partner, one with *KLHL7*, and one with *CDC42BPB*). In those 13 gangliogliomas lacking identifiable *BRAF* alteration, nine contained other genetic alterations predicted to activate the MAP kinase signaling pathway. Two harbored *KRAS* p.Q61K hotspot mutation, one harbored an in-frame *ERC2*-*RAF1* gene fusion, one harbored a hotspot missense mutation (p.N546K) in the kinase domain of *FGFR1*, one harbored an in-frame *FGFR1*-*TACC1* gene fusion, one harbored a mutation affecting the exon 17 splice acceptor sequence of the *FGFR2* gene, and two harbored in-frame *FGFR2* gene fusions (one with *INA* as the fusion partner and the other with *KIAA1598*). One patient with a clinical diagnosis of neurofibromatosis type 1 harbored a germline heterozygous frameshift mutation in the *NF1* gene with somatic loss of the remaining wildtype allele in the tumor. These genetic alterations involving *BRAF*, *KRAS*, *RAF1*, *NF1*, *FGFR1*, and *FGFR2* were mutually exclusive (i.e. no tumor harbored any two of these variants simultaneously). In total, 36 of the 40 tumors (90%) were identified to harbor a genetic alteration predicted to cause activation of the MAP kinase signaling pathway. Among the remaining four tumors, three did not contain identifiable pathogenic alterations, and one epilepsy-associated ganglioglioma in the temporal lobe of a young child (SF-GG-37) was found to harbor a novel *ABL2*-*GAB2* gene fusion predicted to result in an in-frame fusion protein containing the entirety of the kinase domain of the encoded Abelson-related protein tyrosine kinase, similar to the *ABL2* fusions that have been described in a subset of pediatric leukemias [32, 34]. Three gangliogliomas with *BRAF* p.V600E mutation had concurrent *CDKN2A* homozygous deletion (SF-GG-3, SF-GG-9, and SF-GG-11) and one of these three tumors additionally harbored a subclonal missense mutation in the *PTEN* tumor suppressor gene (SF-GG-3). Otherwise, no additional pathogenic mutations, fusions, amplifications, or deletions were identified in any of the 40 gangliogliomas. As such, the *BRAF*, *KRAS*, *RAF1*, *NF1*, *FGFR1*, or *FGFR2* variants were the solitary pathogenic alteration identified in 33 cases (83%). No tumors harbored pathogenic alterations affecting the *IDH1*, *IDH2*, *H3F3A*, *HIST1H3B*, *HIST1H3C*, *SETD2*, *TP53*, *ATRX*, *TERT* (including promoter region), *CIC*, *FUBP1*, *MYB*, *MYBL1*, *EGFR*, *PDGFRA*, *MET*, *PIK3CA*, *PIK3R1*, *MAP2K1*, *PRKCA*, *BCOR*, *BCORL1*, *NTRK1*, *NTRK2*, *NTRK3*, *ALK*, *RELA*, or *NF2* genes.

Chromosomal copy number analysis revealed no chromosomal gains, losses, or focal amplifications or deletions in 26 of the gangliogliomas (Additional file 1: Table S7). Among the other 14 cases, the quantity of chromosomal aberrations per tumor ranged from one to ten. In the majority of cases, chromosomal copy number changes were limited to gains and losses of whole chromosomes or chromosome arms, without focal gains or losses. No focal amplifications or homozygous deletions were identified other than the three gangliogliomas with focal *CDKN2A* homozygous deletion. Tumor SF-GG-35 demonstrated multiple regions of segmental loss involving chromosome 10 (containing the *FGFR2* and *INA* loci) consistent with the process of chromosome shattering that has been termed chromothripsis, which was the likely mechanism of generating the *FGFR2-INA* gene fusion seen in this tumor. Recurrent chromosomal copy number changes in this cohort included trisomy 7 (containing the *BRAF* locus) seen in six tumors, trisomy 5 seen in five tumors, trisomy 12 seen in three tumors, monosomy 9 seen in three tumors, and monosomy 1p seen in three tumors. Four of the six tumors with trisomy 7 were those harboring *BRAF* alterations and likely involved gain of the mutant or fused allele. All three of the tumors with monosomy 9 were those harboring a focal deletion event involving the remaining copy of chromosome 9p21 including the *CDKN2A* gene, resulting in homozygous/biallelic deletion. All three of the tumors with monosomy 1p were located in the cerebral hemispheres of adults and harbored *BRAF* p.V600E mutation (SF-GG-07, SF-GG-11, and SF-GG-13).

### Association of genetic alterations with clinical and imaging features

The age at initial diagnosis was not significantly different among patients with gangliogliomas stratified by *BRAF* p.V600E mutation versus other *BRAF* alteration, any *BRAF* alteration versus *BRAF* wildtype status, or any *BRAF* alteration versus *FGFR* alteration (Table 2). Regarding location, all *FGFR* altered gangliogliomas were located in the cerebral hemispheres, whereas *BRAF* altered tumors were located throughout the neuraxis. The two thalamic gangliogliomas both harbored *BRAF* p.V600E mutation, three of the four cerebellar gangliogliomas harbored *BRAF* p.V600E mutation, and two of three gangliogliomas centered in the spinal cord harbored *KIAA1549*-*BRAF* fusion. The remaining cerebellar and spinal cord tumors lacked identifiable pathogenic alterations. The two tumors harboring *BRAF* fusion with partners other than *KIAA1549* were both located in the cerebral hemispheres. All tumors with variant *BRAF* mutations, *KRAS* mutation, *RAF1* fusion, *NF1* mutation, and *FGFR* alterations were located in the cerebral hemispheres. Imaging features including tumor size, presence of a cystic component, circumscription, and contrast enhancement did not show significant correlation with underlying genetic alterations (Table 2 and Additional file 1: Table S3).

**Table 2:** Clinical, radiographic, and histologic features of 40 gangliogliomas stratified by genetic alterations

Clinicopathologic features		BRAF V600E (*n* = 18)	BRAF other alteration (*n* = 9)	BRAF any alteration (*n* = 27)	BRAF wildtype (*n* = 13)	FGFR alteration (*n* = 5)	Total cohort (*n* = 40)
Age (years), median (range)		15 (3–63)	17 (5–41)	15 (3–63)	32 (0–59)	35 (7–59)	21 (0–63)
Male: Female		13:5	2:7	15:12	8:5	3:2	23:17
Location:	Cerebrum	13 (72%)	7 (78%)	20 (74%)	11 (85%)	5 (100%)	31 (78%)
	Cerebellum	3 (17%)	0 (0%)	3 (11%)	1 (8%)	0 (0%)	4 (10%)
	Thalamus	2(11%)	0 (0%)	2 (7%)	0 (0%)	0 (0%)	2 (5%)
	Spinal cord	0 (0%)	2 (22%)	2 (7%)	1 (8%)	0 (0%)	3 (8%)
Imaging features^1^							
Size (cm), median (range)		3.1 (2.0–5.9)	5.1 (1.8–7.1)	3.6 (1.8–7.1)	2.9 (1.3–16.0)	4.8 (1.3–9.6)	3.4 (1.3–16.0)
Cystic component		9/11 (82%)	6/8 (75%)	15/19 (79%)	8/10 (80%)	3/4 (75%)	23/29 (79%)
Well-circumscribed		3/11 (27%)	5/8 (63%)	8/19 (42%)	5/10 (50%)	2/4 (50%)	13/29 (45%)
Histologic features							
Glial component:	Oligodendroglial	0 (0%)	0 (0%)	0 (0%)	3 (23%)	3 (60%)^2^	3 (8%)
	Astrocytic	18 (100%)	9 (100%)	27 (100%)	10 (77%)	2 (40%)	37 (92%)
Eosinophilic granular bodies		13 (72%)	6 (67%)	19 (70%)	8 (62%)	3 (60%)	27 (68%)
Rosenthal fibers		1 (6%)	1 (11%)	2 (7%)	4 (31%)	1 (20%)	6 (15%)
Calcifications		9 (50%)	4 (44%)	13 (48%)	6 (46%)	3 (60%)	19 (48%)
Perivascular lymphocytes		11 (61%)	8 (89%)	19 (70%)	4 (31%)	1 (20%)	23 (58%)

^1^Based on review of those cases (*n* = 29) with available pre-operative imaging studies

^2^Statistically significant difference (*p* = 0.001) between FGFR-altered tumors versus FGFR-wildtype tumors displaying oligodendroglial glial component (3/5 versus 0/35)

### Association of genetic alterations with histologic features

**Fig. 2:**
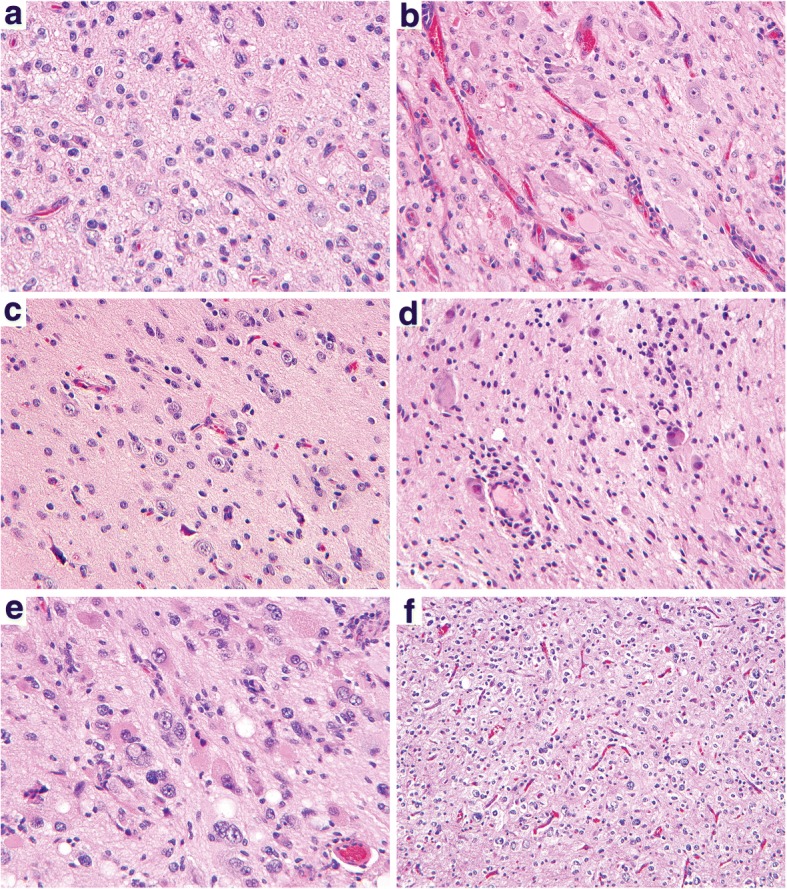
Histologic features of gangliogliomas with different genetic alterations in the MAP kinase signaling pathway. **a** Ganglioglioma in the temporal lobe of a 27 year old man with *BRAF* p.V600E mutation (SF-GG-08). **b** Ganglioglioma in the occipital lobe of a 14 year old boy with *BRAF* p.V600E mutation and *CDKN2A* homozygous deletion (SF-GG-03). **c** Ganglioglioma in the temporal lobe of a 23 year old woman with *BRAF* p.L505delinsLEYLS mutation (SF-GG-22). **d** Ganglioglioma in the spinal cord of a 5 year old girl with *KIAA1549*-*BRAF* gene fusion (SF-GG-24). **e** Ganglioglioma in the frontal lobe of a 48 year old man with *ERC2*-*RAF1* gene fusion (SF-GG-28). **f** Ganglioglioma in the temporal lobe of a 7 year old boy with FGFR2-KIAA1598 gene fusion (SF-GG-34)

All three gangliogliomas with a glial component showing oligodendroglial morphology harbored *FGFR* alterations (Fig. 2 and Additional file 2: Figure S1). However, the other two gangliogliomas with *FGFR* alterations had a glial component with astrocytic morphology. All *BRAF*, *KRAS*, *NF1*, and *RAF1* altered tumors had a glial component with astrocytic morphology. Except for the morphology of the glial component, none of the other histologic features including presence/absence of eosinophilic granular bodies, Rosenthal fibers, calcifications, and perivascular lymphocytes showed a significant correlation with underlying genetic alterations (Table 2 and Additional file 1: Table S4).

### Association of genetic alterations with disease recurrence or progression

**Fig. 3:**
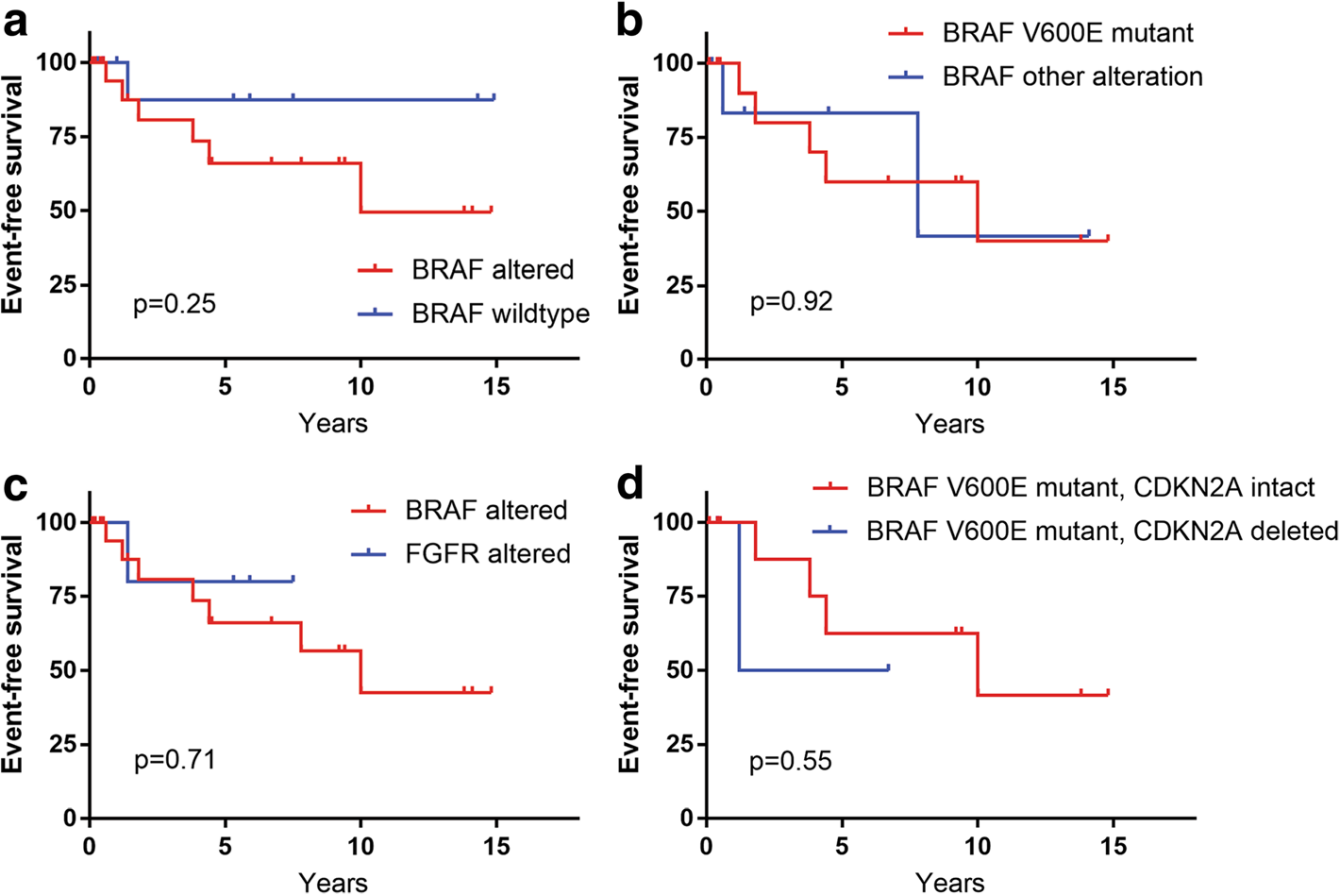
Event-free survival of the 40 patients with ganglioglioma stratified by genetic alterations. **a-d** Shown are Kaplan-Meier curves of event-free survival (either recurrence after gross total resection or disease progression after subtotal resection) from the ganglioglioma cohort stratified by *BRAF* altered versus *BRAF* wildtype (**a**), *BRAF* V600E mutant versus other *BRAF* alteration (**b**), *BRAF* altered versus *FGFR* altered (**c**), and *BRAF* V600E mutant with *CDKN2A* intact versus *BRAF* V600E mutant with *CDKN2A* homozygous deletion (**d**). *p* values were calculated by Log-rank (Mantel-Cox) test

In the two gangliogliomas that recurred after gross total resection, sequencing analysis that was performed on the recurrent tumors demonstrated *BRAF* p.R506delinsRVLR mutation as the solitary pathogenic alteration without chromosomal copy number alterations in one case. The other demonstrated *BRAF* p.V600E mutation, *CDKN2A* homozygous deletion, a subclonal missense mutation in the *PTEN* tumor suppressor gene, and only two chromosomal copy number aberrations (gain of distal 3q and loss of 9). In the four gangliogliomas that showed tumor progression after subtotal resection, three harbored *BRAF* p.V600E mutation as the solitary pathogenic alteration without chromosomal copy number aberrations, and the fourth tumor harbored *CDC42BPB*-*BRAF* gene fusion. In the two gangliogliomas that progressed after initial resection of unknown extent, sequencing analysis that was performed on the recurrent tumors demonstrated *FGFR2*-*INA* fusion in one and *BRAF* p.V600E mutation in the other. Event-free survival of the patient cohort stratified by *BRAF* altered versus *BRAF* wildtype status, *BRAF* V600E mutant versus other *BRAF* alteration, *BRAF* altered versus *FGFR* altered, and *BRAF* V600E mutant/*CDKN2A* intact versus *BRAF* V600E mutant/*CDKN2A* deleted was assessed (Fig. 3). No significant differences in event-free survival were found based on underlying genetic alterations in this cohort.

## Discussion

This study reveals that ganglioglioma is genetically defined by alterations that activate the MAP kinase signaling pathway in the vast majority of cases, either via *BRAF* p.V600E mutation or a spectrum of other genetic alterations including alternative *BRAF* mutations or fusions, *RAF1* fusion, *KRAS* mutation, *NF1* mutation, or *FGFR* mutations or fusions. In the majority of cases, the genetic alteration within the MAP kinase pathway was the solitary genetic alteration identified, with few (if any) chromosomal copy number changes, indicating that most gangliogliomas are genetically simple tumors. As such, gangliogliomas are genetically similar to pilocytic astrocytoma, DNET, rosette-forming glioneuronal tumor (RGNT), PLNTY, and MVNT. Gangliogliomas more frequently harbor *BRAF* p.V600E mutation or other variant *BRAF* mutations than pilocytic astrocytomas, which most commonly harbor *KIAA1549*-*BRAF* fusion [19, 30, 38]. One study of posterior fossa and spinal cord gangliogliomas with a glial component resembling pilocytic astrocytoma found that a subset harbored *KIAA1549*-*BRAF* fusion that the authors referred to “pilocytic astrocytoma with focal gangliocytic differentiation” [16]. The two gangliogliomas in our cohort with *KIAA1549*-*BRAF* fusion were both located in the spinal cord of children and demonstrated numerous dysmorphic ganglion cells throughout the entirety of the tumor, indicating that classic gangliogliomas can also harbor *KIAA1549*-*BRAF* fusion. While the majority of DNETs and RGNTs harbor *FGFR1* mutation or rearrangement [14, 33, 37], this is only present in a small subset of pathologically-confirmed gangliogliomas. The recently described entity PLNTY has been reported to harbor either *FGFR* fusions or *BRAF* V600E mutation [17], which overlaps with the genetic alterations observed in gangliogliomas. Our recent genetic analysis revealed that MVNTs harbor frequent *MAP2K1* exon 2 mutations or small in-frame deletions, as well as *BRAF* mutations other than V600E [28]; however, MAP kinase pathway activation in gangliogliomas appears to occur independently of *MAP2K1* alterations.

Pleomorphic xanthoastrocytoma is a circumscribed glial neoplasm that is genetically characterized by concurrent *CDKN2A* homozygous deletion and *BRAF* p.V600E mutation (or less commonly *BRAF* or *RAF1* fusion) [29, 38]. However, our study shows that a small subset of pathologically-confirmed gangliogliomas can harbor this identical combination of *CDKN2A* homozygous deletion and *BRAF* p.V600E mutation, indicating that this genetic pattern is not entirely specific to pleomorphic xanthoastrocytomas. Another recently described tumor entity is diffuse leptomeningeal glioneuronal tumor (also referred to as disseminated oligodendroglioma-like leptomeningeal neoplasm), which is genetically characterized by the combination of monosomy 1p and *KIAA1549*-*BRAF* fusion [35]. Three gangliogliomas in our cohort harbored the combination of monosomy 1p and *BRAF* p.V600E mutation, all of which were intraparenchymal tumors located in the cerebral hemispheres of adults and did not display widespread leptomeningeal dissemination. Of note, a series of intramedullary low-grade glioneuronal tumors of the spinal cord in children harboring *BRAF* fusion and monosomy 1p without diffuse leptomeningeal spread was recently reported [8]. The relationship of these pediatric spinal tumors and our adult cerebral gangliogliomas harboring the combination of monosomy 1p and *BRAF* p.V600E mutation is uncertain.

The genetic profile of ganglioglioma appears to be distinct from several glial and glioneuronal neoplasms. No *PRKCA* fusions or kinase domain mutations were identified in any of the cases, suggesting that gangliogliomas are genetically distinct from the majority of papillary glioneuronal tumors and chordoid gliomas [3, 15]. No *IDH1*, *IDH2*, *TP53*, *ATRX*, *TERT* promoter, *CIC*, or *FUBP1* mutations were identified in any of the cases, suggesting that gangliogliomas are genetically distinct from the majority of diffuse lower-grade gliomas in adults (both astrocytomas and oligodendrogliomas) [4]. No *MYB* or *MYBL1* rearrangements were identified in any of the cases, suggesting that gangliogliomas are genetically distinct from the majority of angiocentric gliomas and pediatric IDH-wildtype diffuse astrocytomas [1, 30, 31, 38]. No *TSC1* or *TSC2* mutations were identified in any of the cases, suggesting that gangliogliomas are also genetically distinct from the majority of subependymal giant cell astrocytomas [5].

Malformations of cortical development, including focal cortical dysplasia, constitute one of the major differential diagnoses for ganglioglioma. Genetic evaluation of sporadic focal cortical dysplasias (not associated with another lesion) has revealed frequent post-zygotic somatic mutations in components of the PI3-kinase-Akt-mTOR signaling pathway, most often involving the *TSC1*, *TSC2*, *AKT3*, *MTOR*, *PIK3CA*, or *PTEN* genes [10, 18, 23–26]. None of the gangliogliomas in this cohort showed genetic alterations in components of this pathway, except for one ganglioglioma that recurred after gross total resection and harbored a subclonal *PTEN* missense mutation (in addition to *BRAF* p.V600E mutation and *CDKN2A* homozygous deletion). This indicates that the *PTEN* mutation was likely acquired during tumor progression and was not the initiating genetic driver. Thus, gangliogliomas appear to be genetically distinct from the majority of sporadic focal cortical dysplasias, which suggests that genetic evaluation may be potentially informative in cortical resection cases that are challenging to classify based on morphologic features.

Four of the gangliogliomas in this cohort harbored recurrent small in-frame insertions at codon 505 or 506 in the β3-αC loop in the kinase domain of *BRAF* (p.L505delinsLEYLS, p.R506delinsRVLR [in two cases], and p.R506delinsRSTQ). Among the 52,519 tumors with *BRAF* mutations currently cataloged in the COSMIC database [version 85 release], only one other tumor (medulloblastoma) with a small in-frame insertion at this site is present. Given this recurrent *BRAF* alteration in a tumor type with frequent MAP kinase pathway activation and low somatic mutation burden, together with a lack of other identifiable alterations in MAP kinase pathway genes in these four tumors, this very likely represents a novel hotspot *BRAF* mutation causing activation of the serine/threonine kinase domain in gangliogliomas.

Four of the gangliogliomas in this cohort lacked identifiable alterations in canonical genes associated with the MAP kinase pathway. These cases may potentially harbor cryptic alterations in MAP kinase genes that were not detectable by this sequencing assay. Alternatively, these tumors may harbor novel molecular alterations and represent rare molecular subtypes of ganglioglioma or other glioneuronal tumors. Indeed, one of these four tumors was identified to harbor a novel *ABL2*-*GAB2* gene fusion. Whether this fusion leads to downstream activation of the MAP kinase pathway similar to most other gangliogliomas, or instead drives proliferation via modulation of other intracellular signaling pathway is unknown.

Our study does not reveal any differences in genetic profile of gangliogliomas that correlate with disease progression or recurrence. This may be due to the small size of the cohort in this study, particularly those with less common variants such as *RAF1* fusion or *KRAS* mutation. However, as the predicted biologic consequence of the less common MAP kinase variants identified in this study is activation of the same Ras-Raf-MEK-ERK signaling pathway as *BRAF* p.V600E mutation, we hypothesize that the specific MAP kinase pathway alteration is unlikely to dictate differences in clinical behavior. Instead, other factors such as tumor location, extent of resection, accompanying genetic alterations, and/or epigenetic differences are more likely to drive the clinical variability in presentation and outcome for patients with ganglioglioma.

## Additional files

Additional file 1: **Tables S1-S7.** (XLSX 44 kb)

Additional file 2: **Figures S1-S2.** (PDF 7457 kb)
