# Supplementary material for: The genetic landscape of ganglioglioma
Source: Acta Neuropathol Commun. 2018 Jun 7;6:47. doi: 10.1186/s40478-018-0551-z (PMC5992851; doi:10.1186/s40478-018-0551-z)

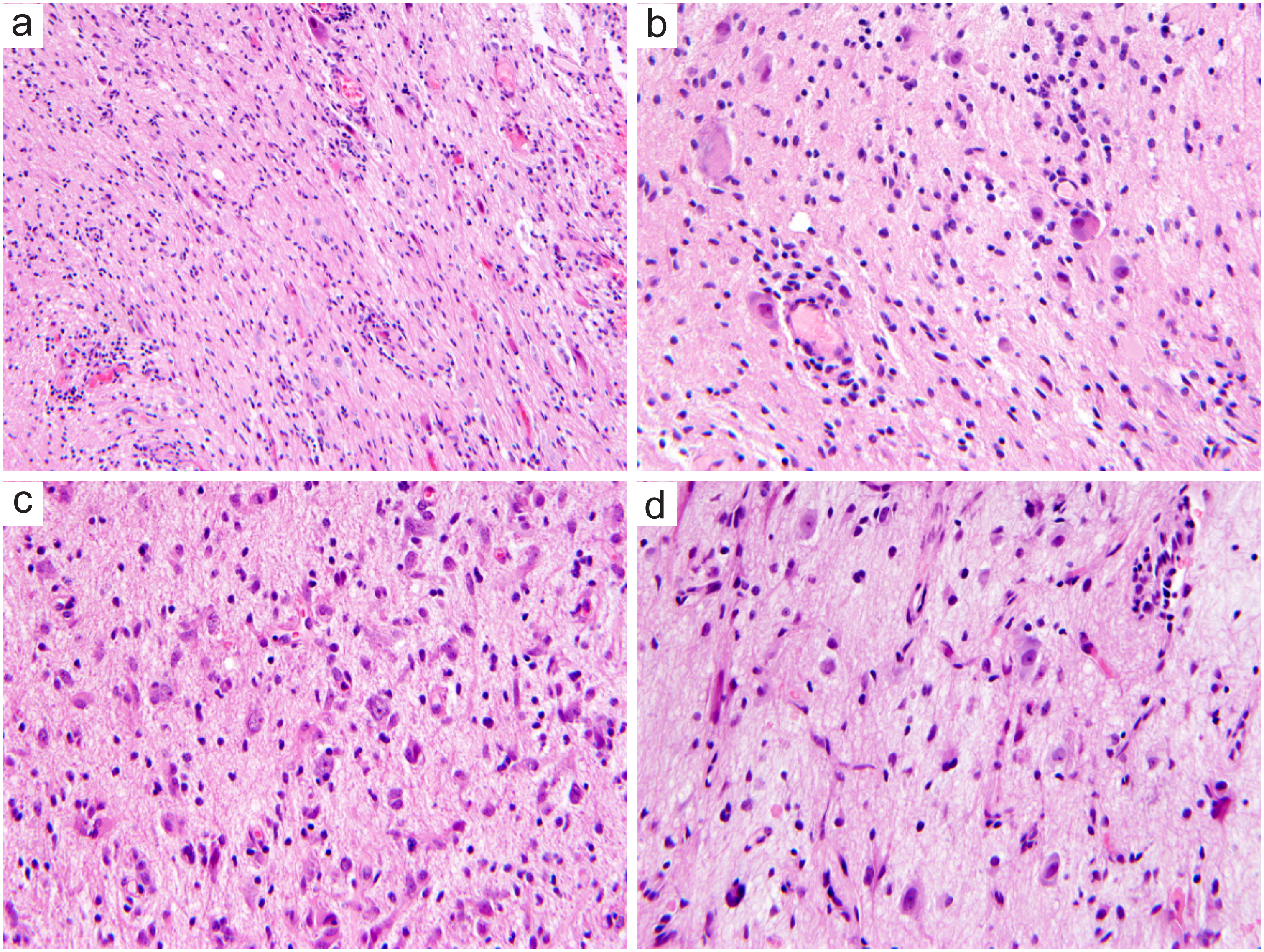


**Supplementary Fig. 1** Histologic features of gangliogliomas with various genetic alterations.
**a,b** Ganglioglioma in the spinal cord of a 5 year old girl with *KIAA1549-BRAF* gene fusion (SF-GG-24).
**c** Ganglioglioma in the temporal lobe of a 9 year old girl with *CDC42BPB-BRAF* gene fusion (SF-GG-26).
**d** Ganglioglioma in the temporal lobe of a 32 year old woman with *KRAS* p.Q61K mutation (SF-GG-29).
**e** Ganglioglioma in the temporal lobe of a 28 year old man with germline *NF1* mutation and somatic loss of the remaining wildtype allele in the tumor (SF-GG-31).
**f** Ganglioglioma in the temporal lobe of 59 year old woman with *FGFR1* p.N546K mutation (SF-GG-33).
**g,h** Ganglioglioma in the temporal lobe of a 7 year old boy with *FGFR2-KIAA1598* gene fusion (SF-GG-34).
**i,j** Ganglioglioma in the frontal lobe of a 2 year old boy with *ABL2-GAB2* gene fusion (SF-GG-37).

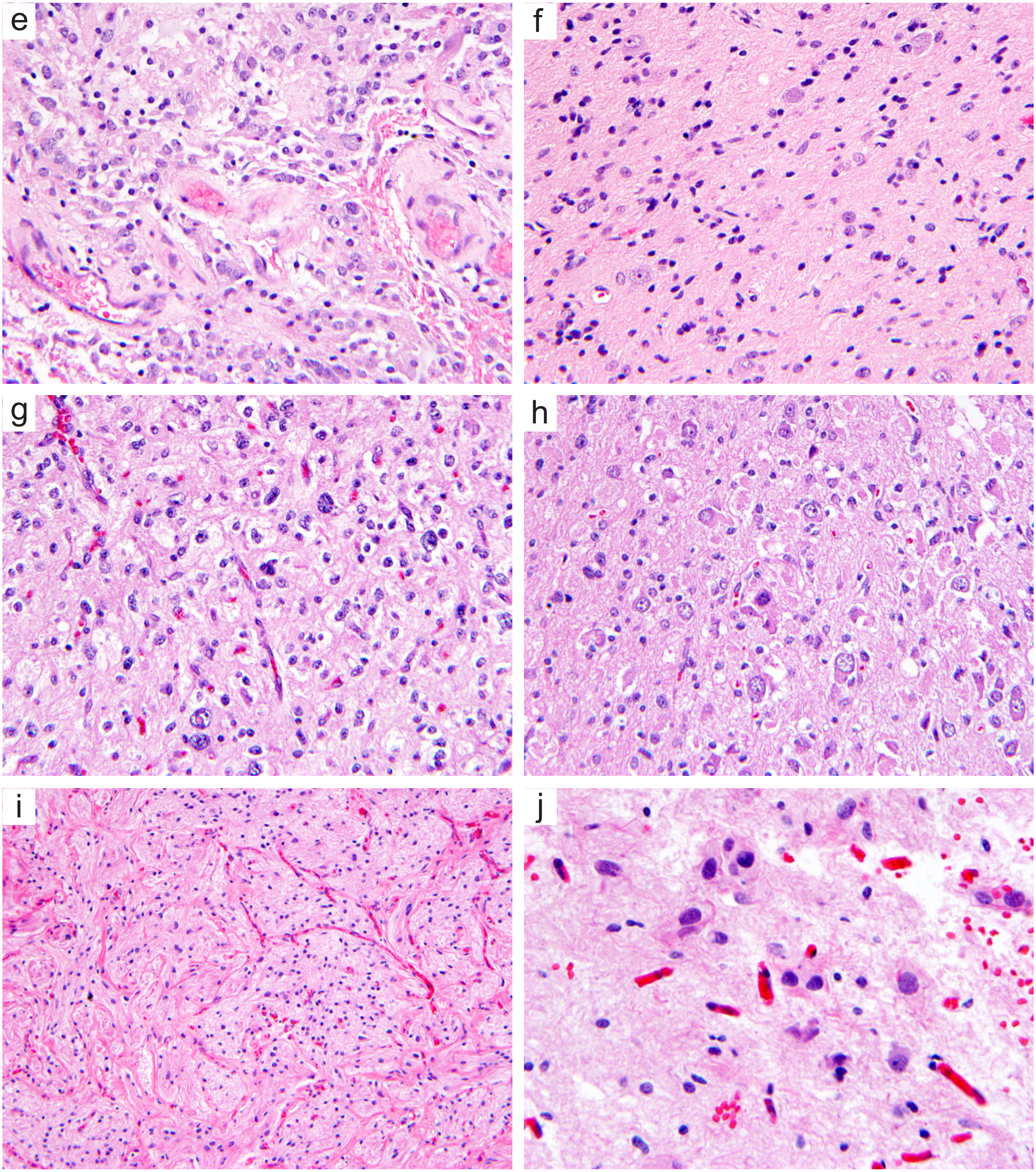
e
f
g
h
i
j

a

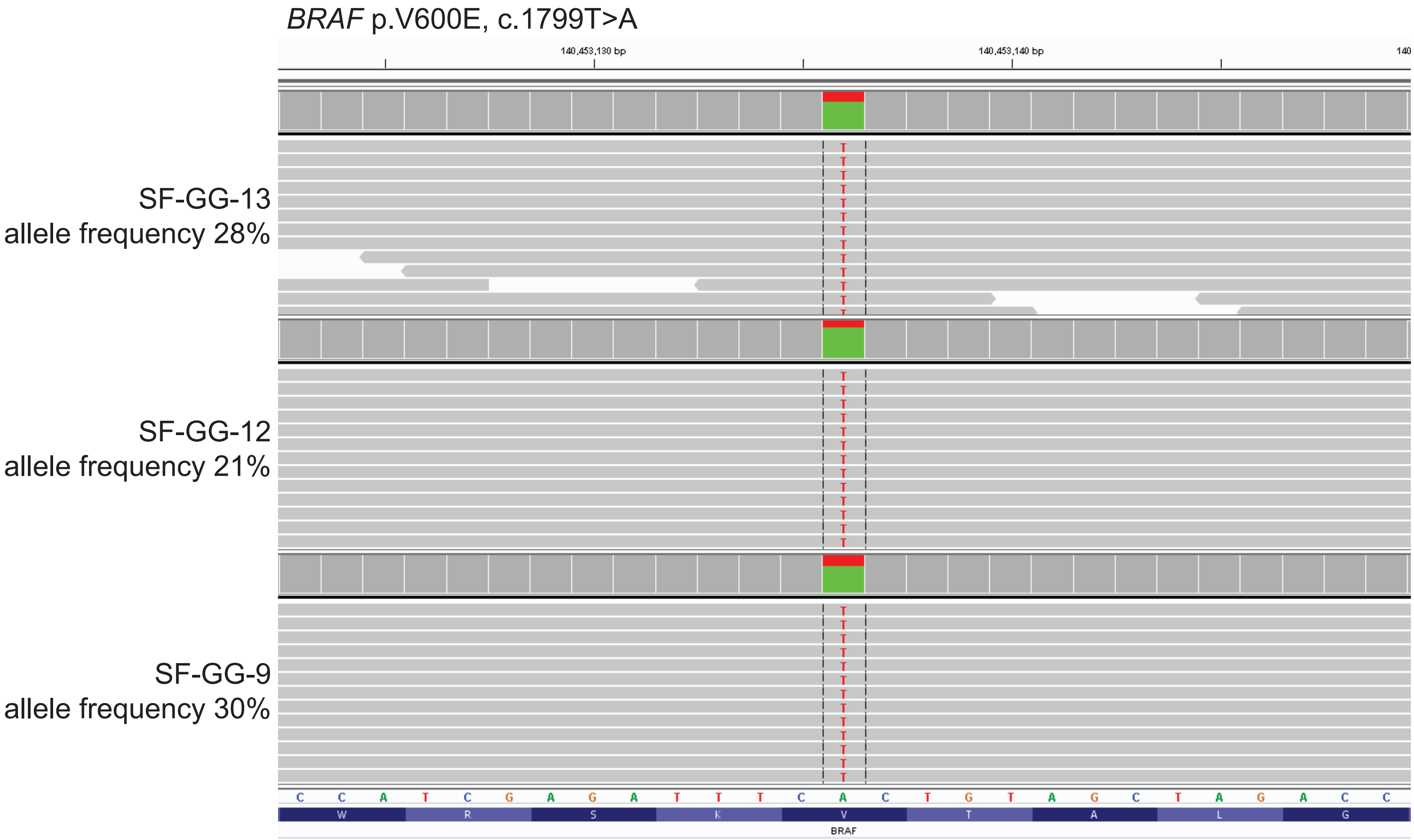


**Supplementary Fig. 2** Snapshots from the Integrated Genome Viewer of genetic alterations identified in the 40 gangliogliomas. **a** Gangliogliomas with *BRAF* p.V600E mutation. **b** Ganglioglioma with *BRAF* p.T599_W604delinsTDG mutation. **c** Gangliogliomas with *BRAF* p.R506delins mutation. **d** Ganglioglioma with *KIAA1549-BRAF* fusion. **e** Ganglioglioma with *CDC42BPB-BRAF* fusion. **f** Ganglioglioma with *KLHL7-BRAF* fusion. **g** Ganglioglioma with *ERC2-RAF1* fusion. **h** Gangliogliomas with *KRAS* p.Q61K mutation. **i** Ganglioglioma in patient with a heterozygous germline *NF1* p.Q514frameshift mutation with somatic loss of the remaining wildtype allele in the tumor. **j** Ganglioglioma with *FGFR1* p.N546K mutation. **k** Ganglioglioma with *FGFR2* exon 17 splice acceptor mutation. **l** Ganglioglioma with *FGFR1-TACC1* fusion. **m** Ganglioglioma with *FGFR2-KIAA1598* fusion. **n** Ganglioglioma with *FGFR2-INA* fusion. **o** Ganglioglioma with *ABL2-GAB2* fusion.

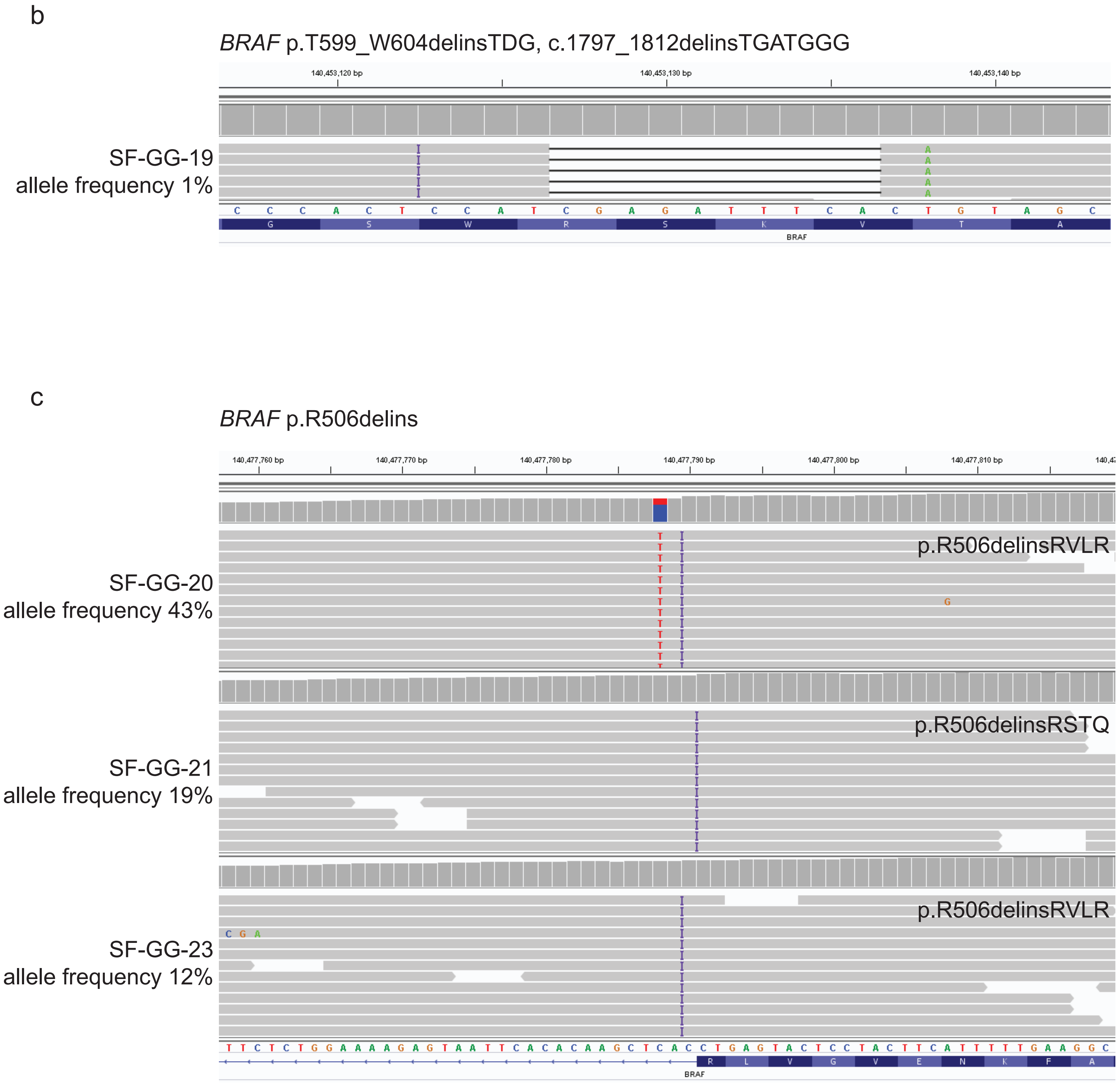
b
BRAF p.T599_W604delinsTDG, c.1797_1812delinsTGATGGG
140,453,120 bp
140,453,130 bp
140,453,140 bp
SF-GG-19
allele frequency 1%
BRAF
c
BRAF p.R506delins
140,477,760 bp
140,477,770 bp
140,477,780 bp
140,477,790 bp
140,477,800 bp
140,477,810 bp
p.R506delinsRVLR
SF-GG-20
allele frequency 43%
p.R506delinsRSTQ
SF-GG-21
allele frequency 19%
p.R506delinsRVLR
SF-GG-23
allele frequency 12%
BRAF

d

*KIAA1549-BRAF* fusion (exons 1-10 of *KIAA1549* to exons 9-18 of *BRAF*)

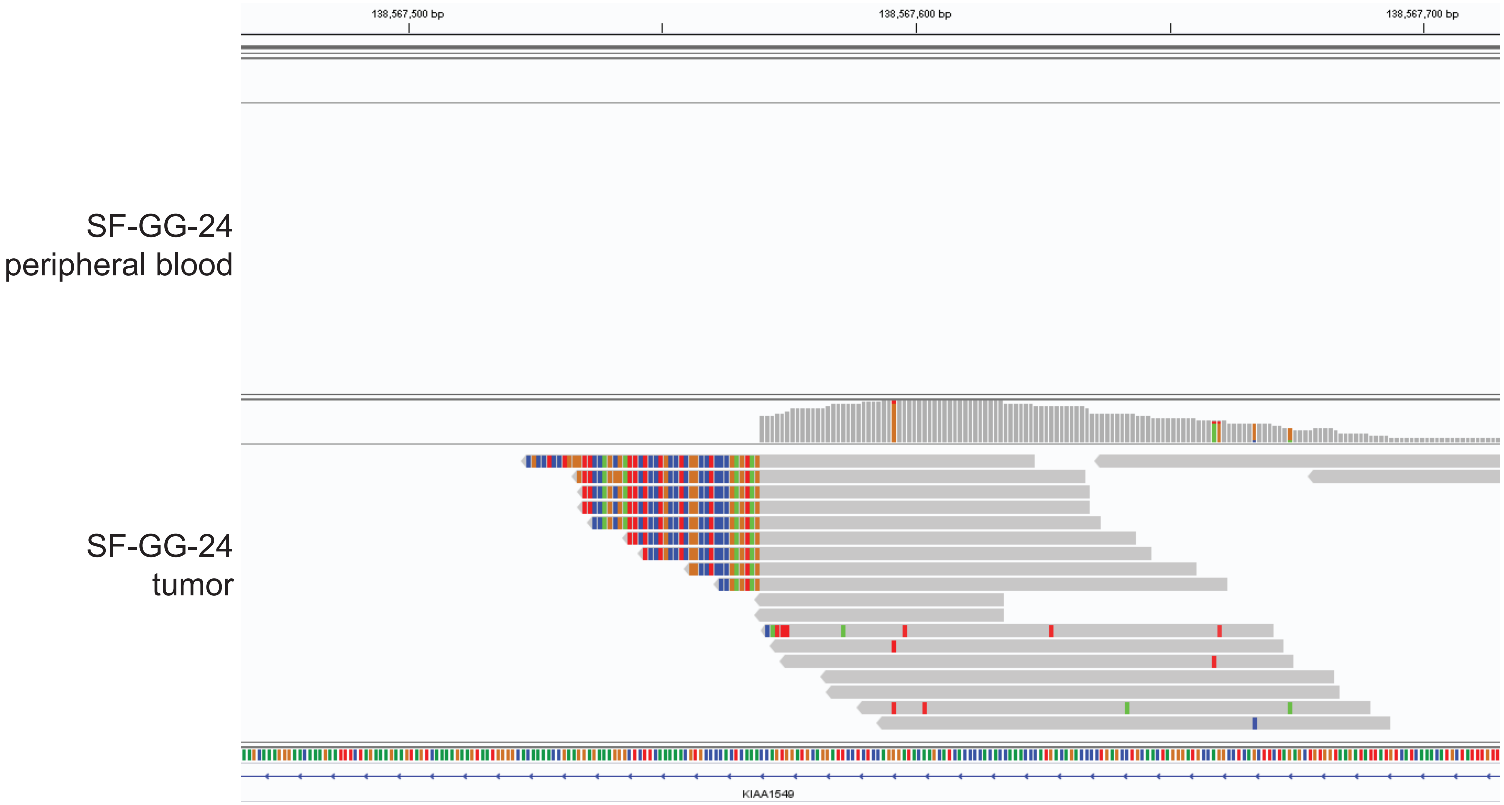

e

*CDC42BPB-BRAF* fusion (exons 1-36 of *CDC42BPB* to exons 9-18 of *BRAF*)

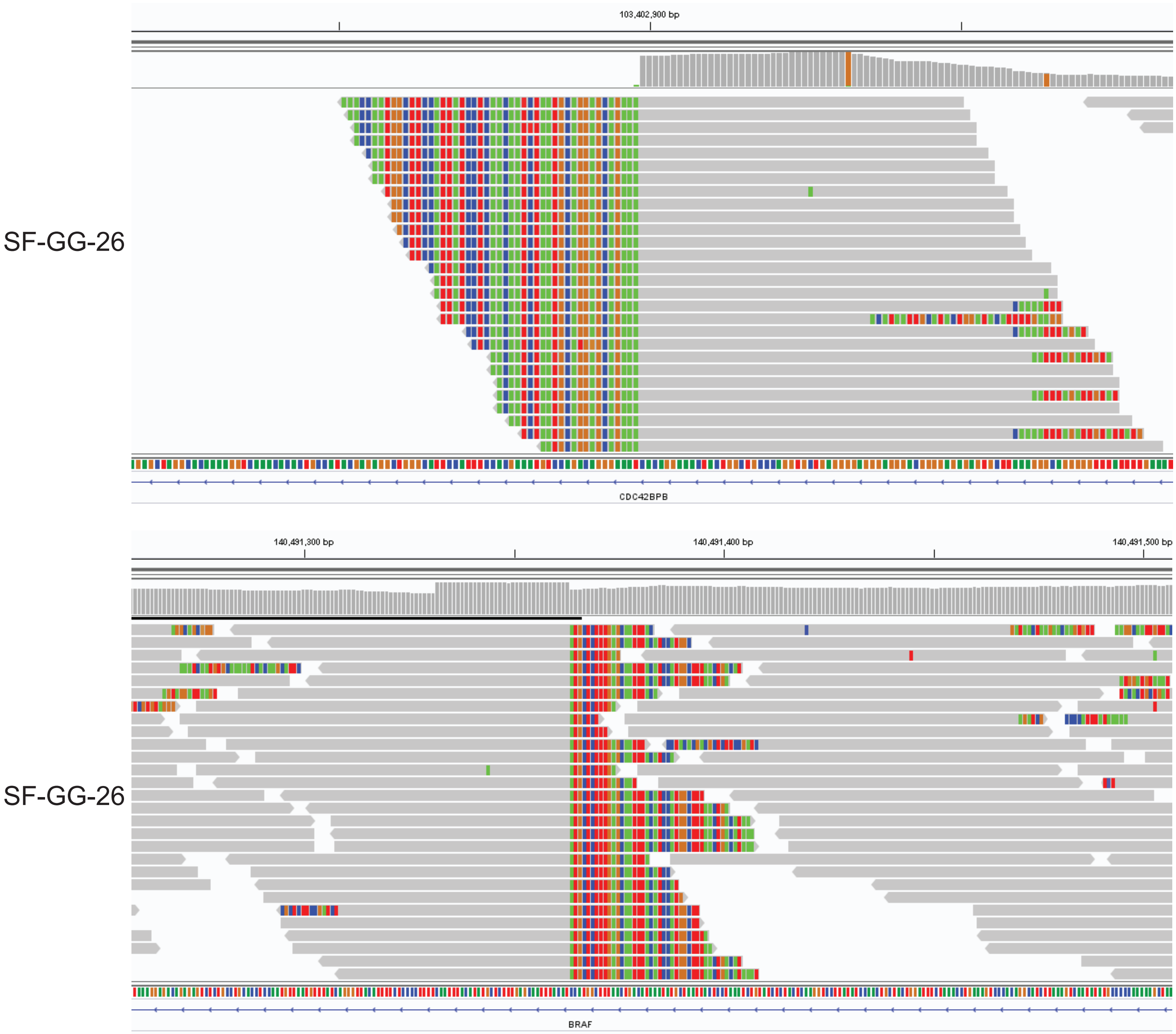

f

*KLHL7-BRAF* fusion (exons 1-4 of *KLHL7* to exons 10-18 of *BRAF*)

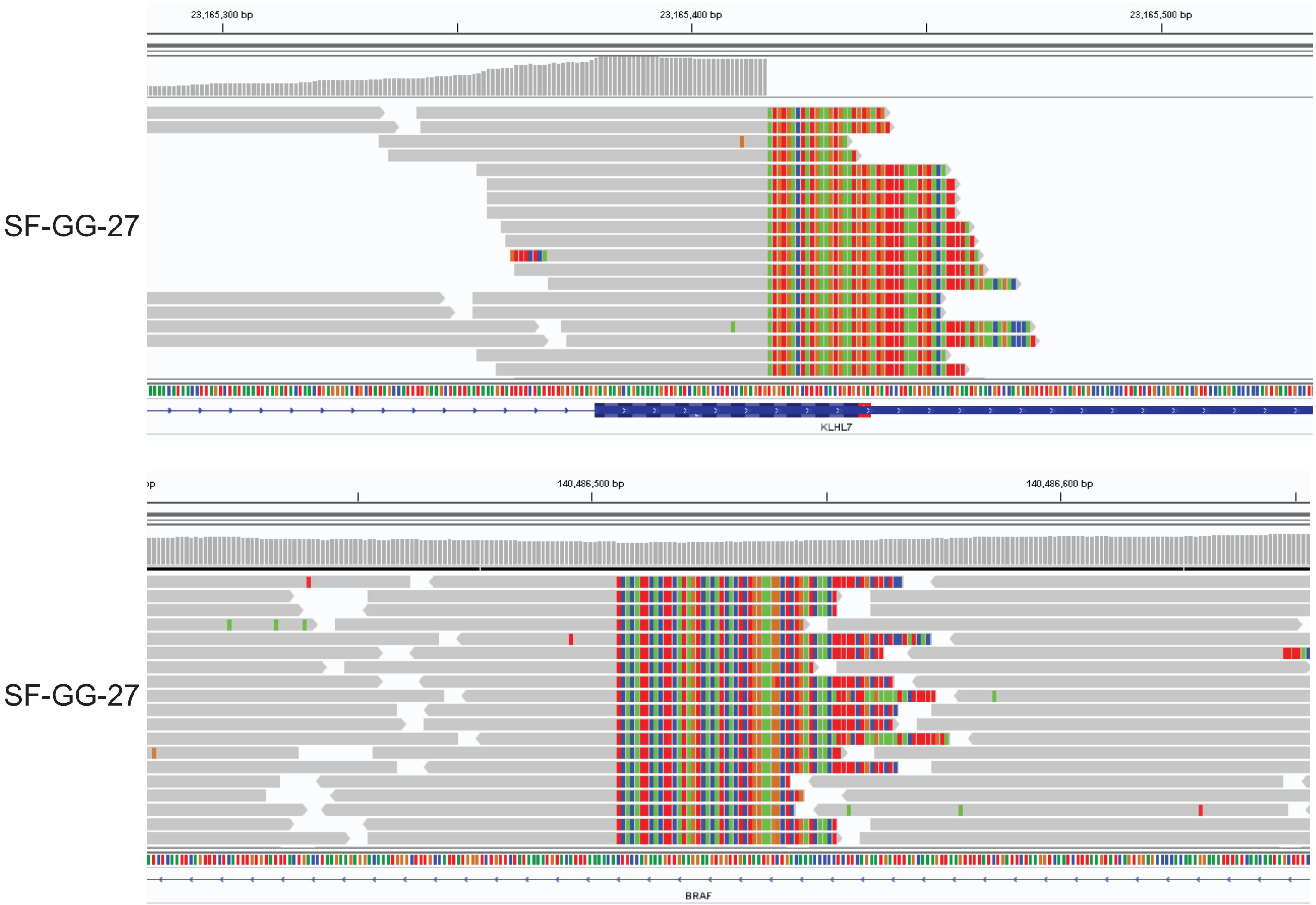

g

*ERC2-RAF1* fusion (exons 1-7 of *ERC2* to exons 8-17 of *RAF1*)

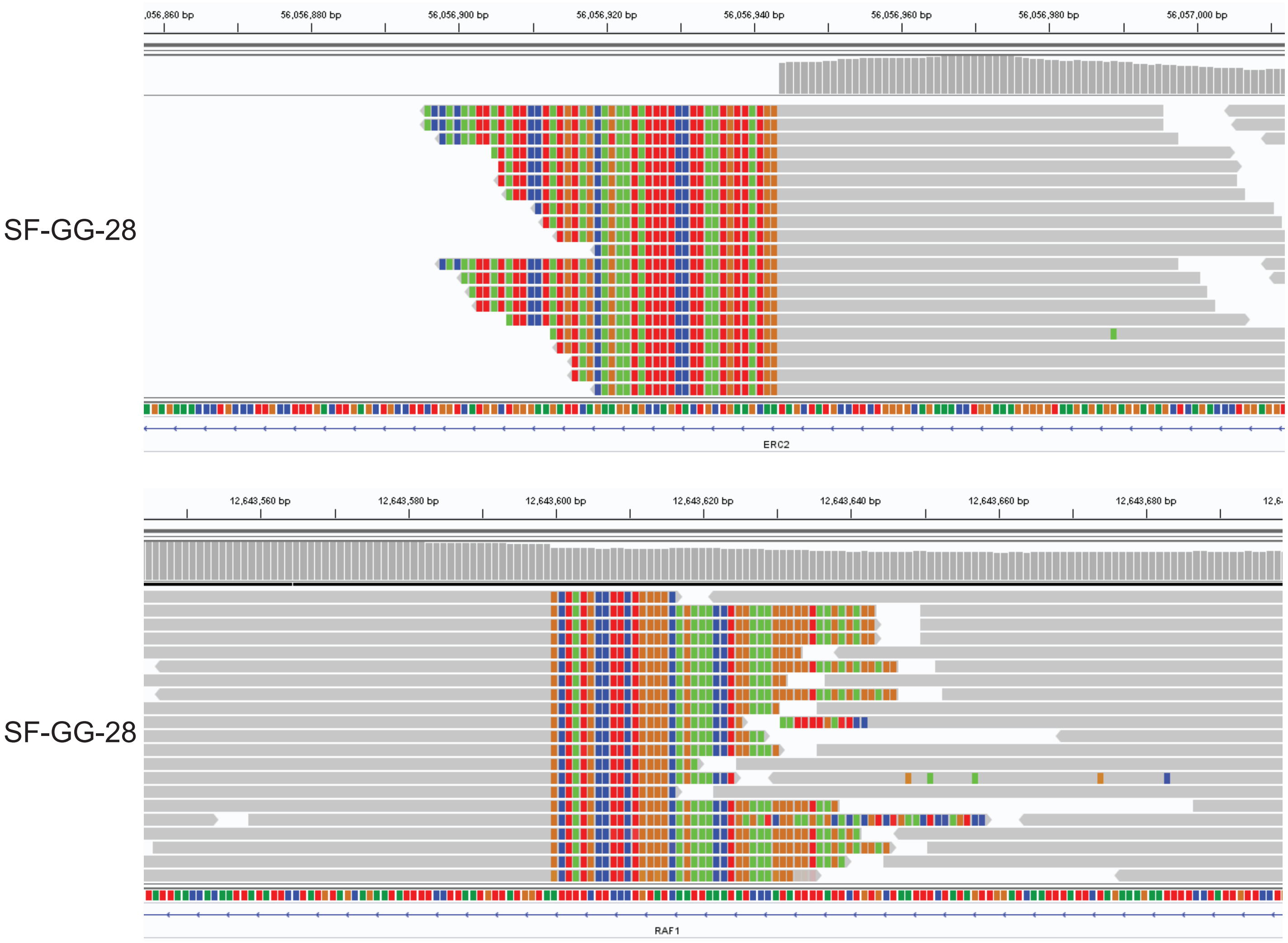

h

*KRAS* p.Q61K, c.180_181delinsCA (SF-GG-29) and c.180_181delinsAA (SF-GG-30)

SF-GG-29
allele frequency 29%

SF-GG-30
allele frequency 20%

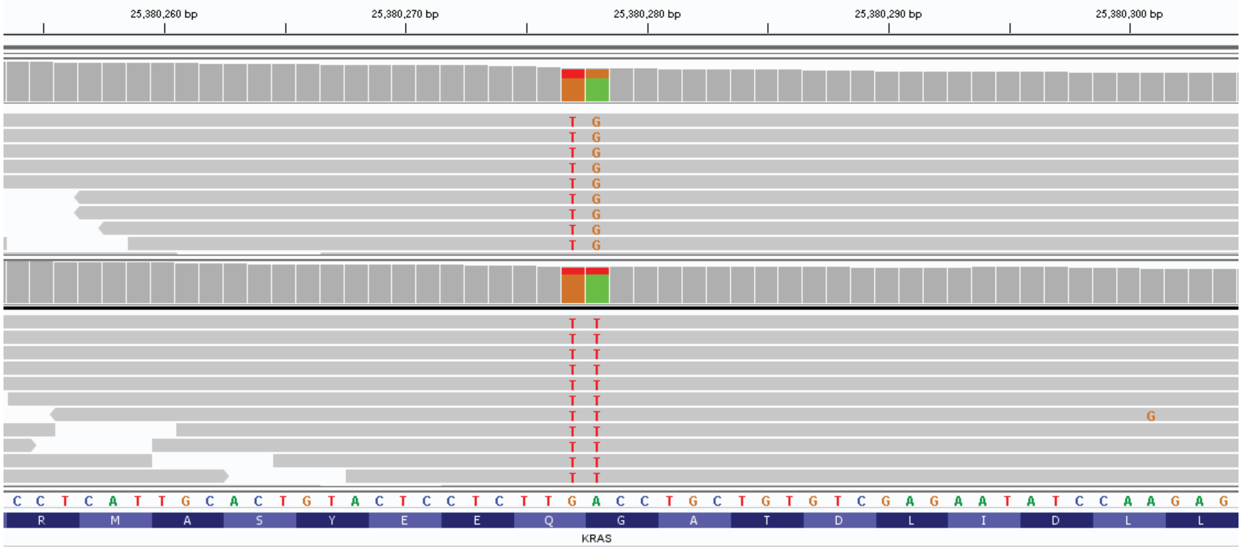


i

*NF1* p.Q514frameshift, c.1541_1542del

SF-GG-31
peripheral blood
allele frequency 51%

SF-GG-31
tumor
allele frequency 83%

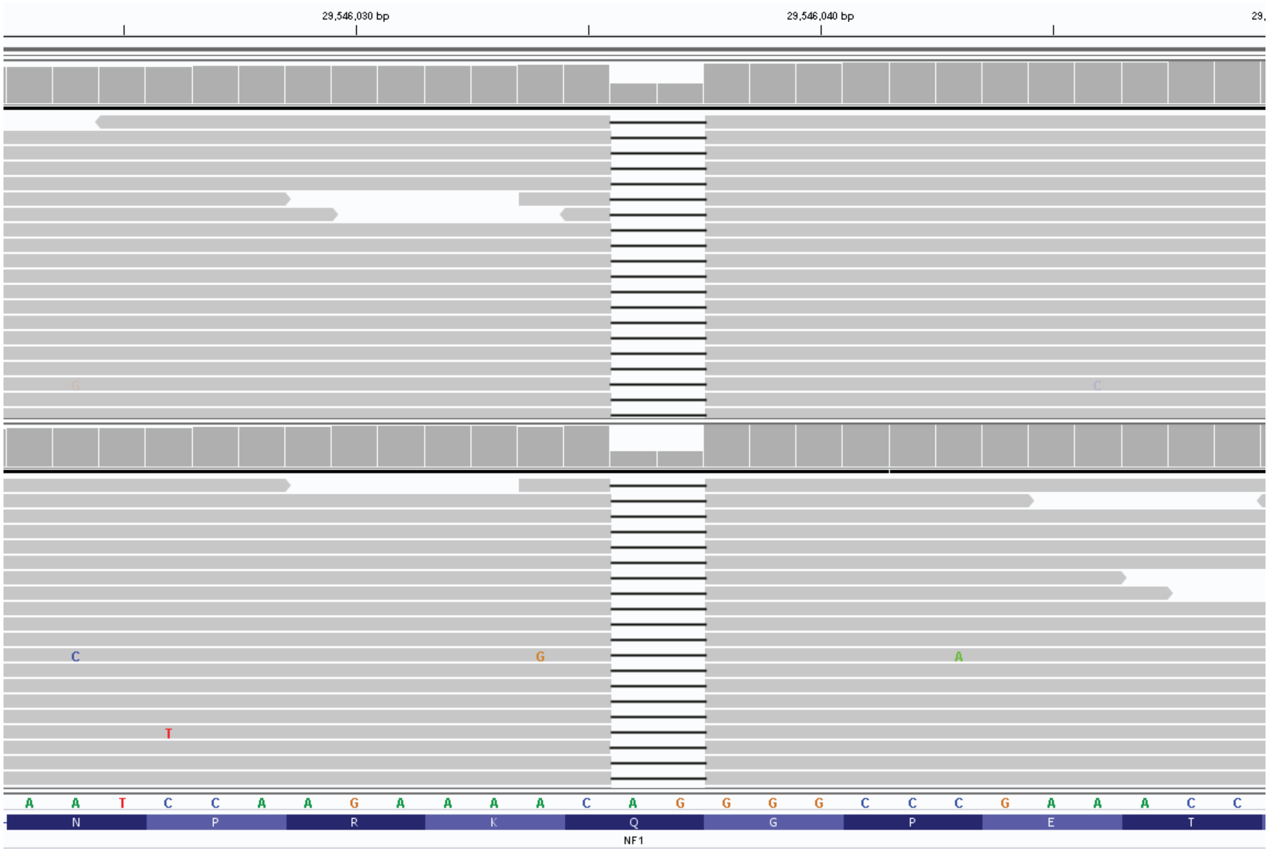

j

*FGFR1* p.N546K, c.1638C>A

SF-GG-33
allele frequency 3%

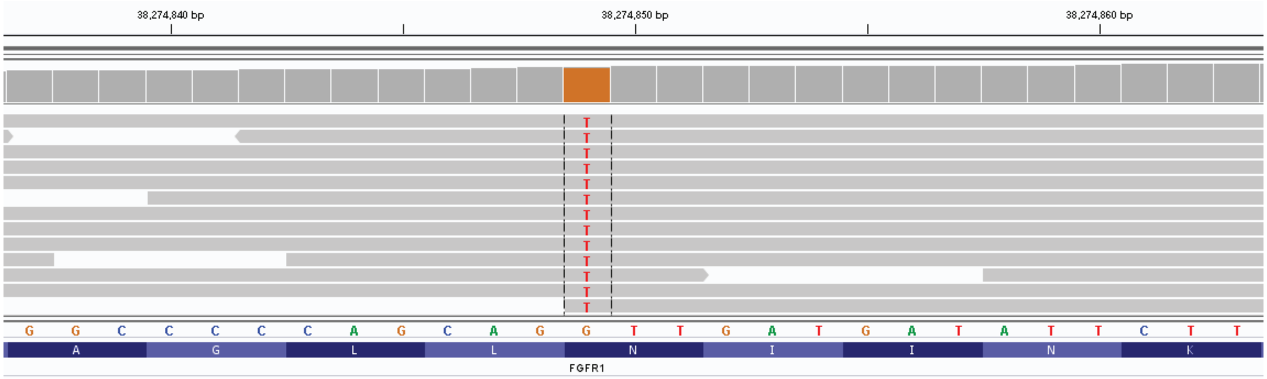


k

*FGFR2* exon 17 splice acceptor, c.2199-2A>T

SF-GG-36
allele frequency 4%

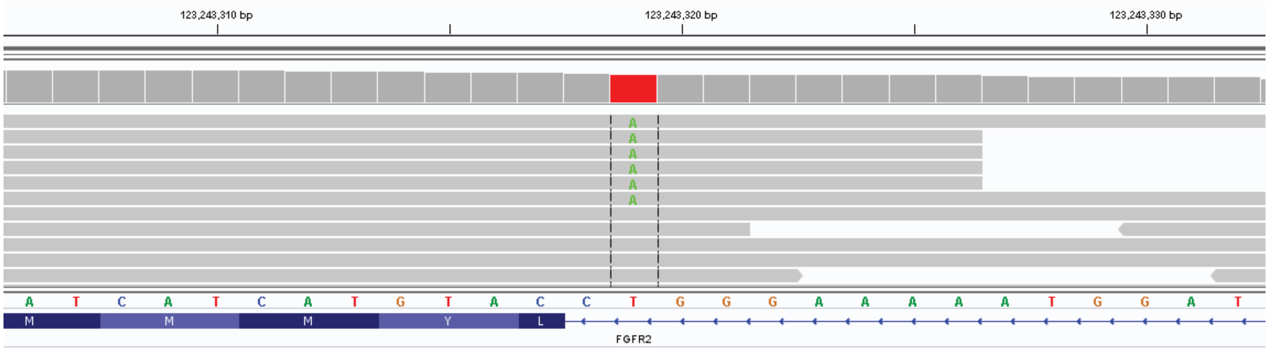

I

*FGFR1-TACC1* fusion (exons 1-18 of *FGFR1* to exons 7-13 of *TACC1*)

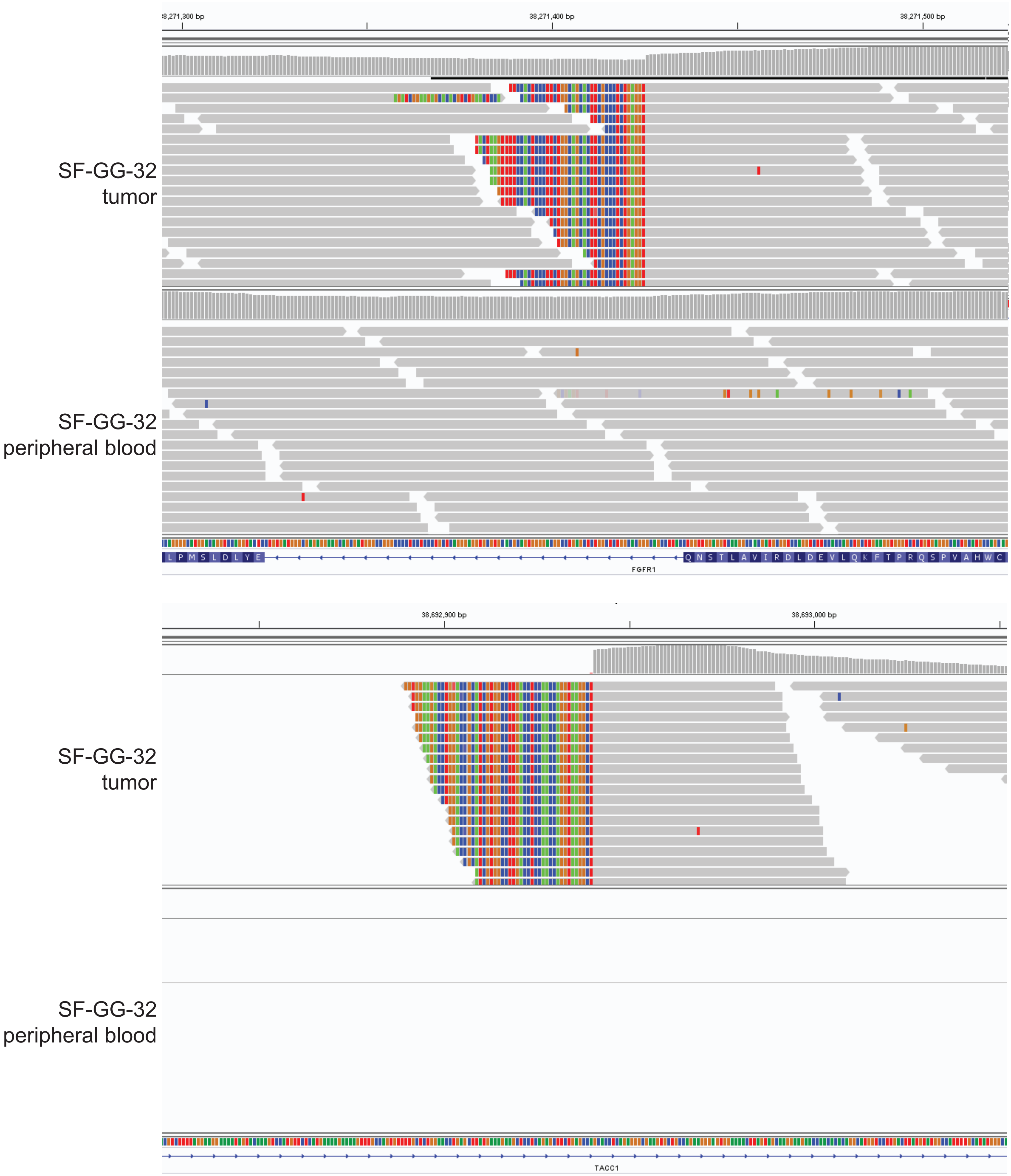

m

*FGFR2-KIAA1598* fusion (exons 1-17 of *FGFR2* to exons 7-17 of *KIAA1598*)

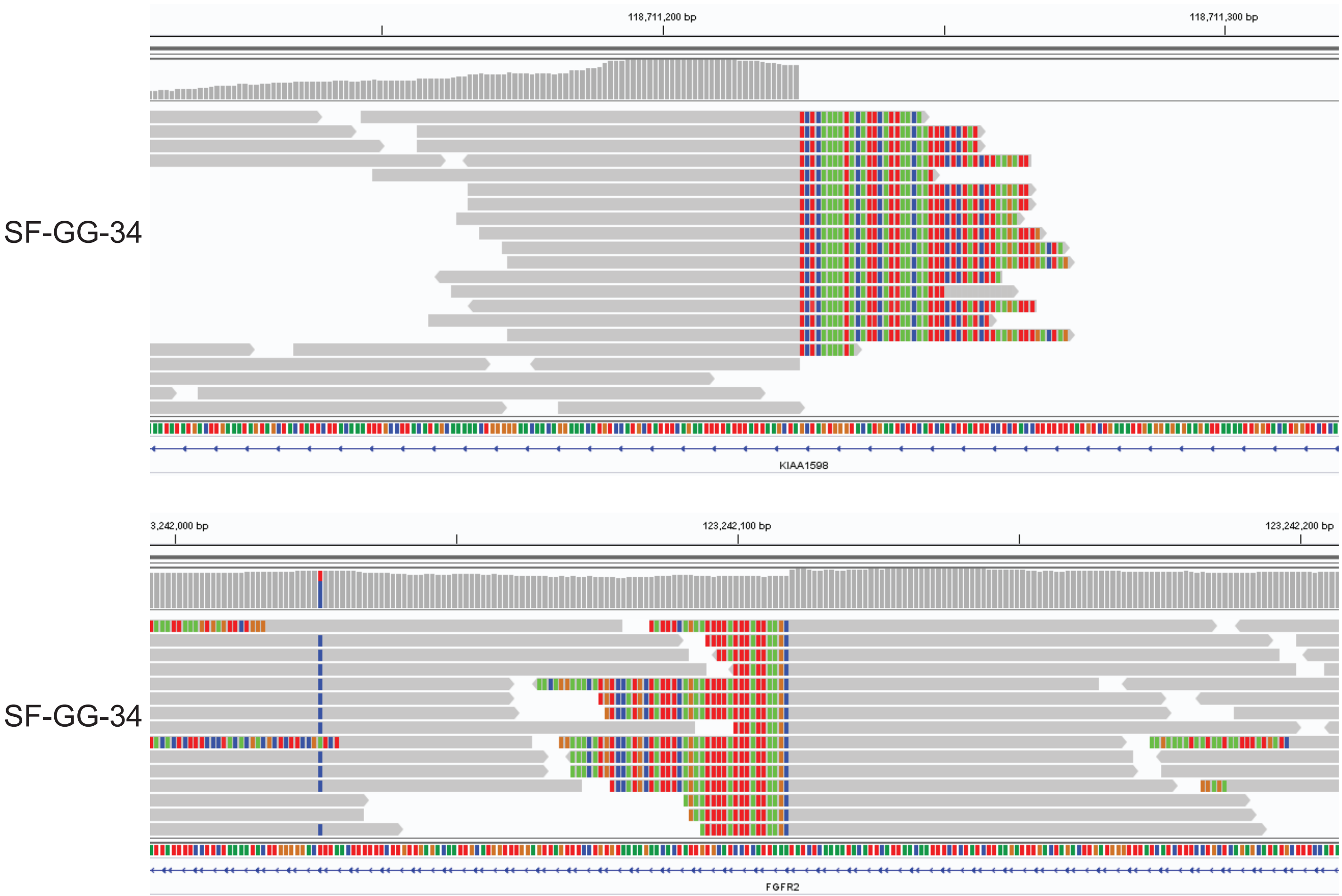

n

*FGFR2-INA* fusion (exons 1-17 of *FGFR2* to exons 2-3 of *INA*)

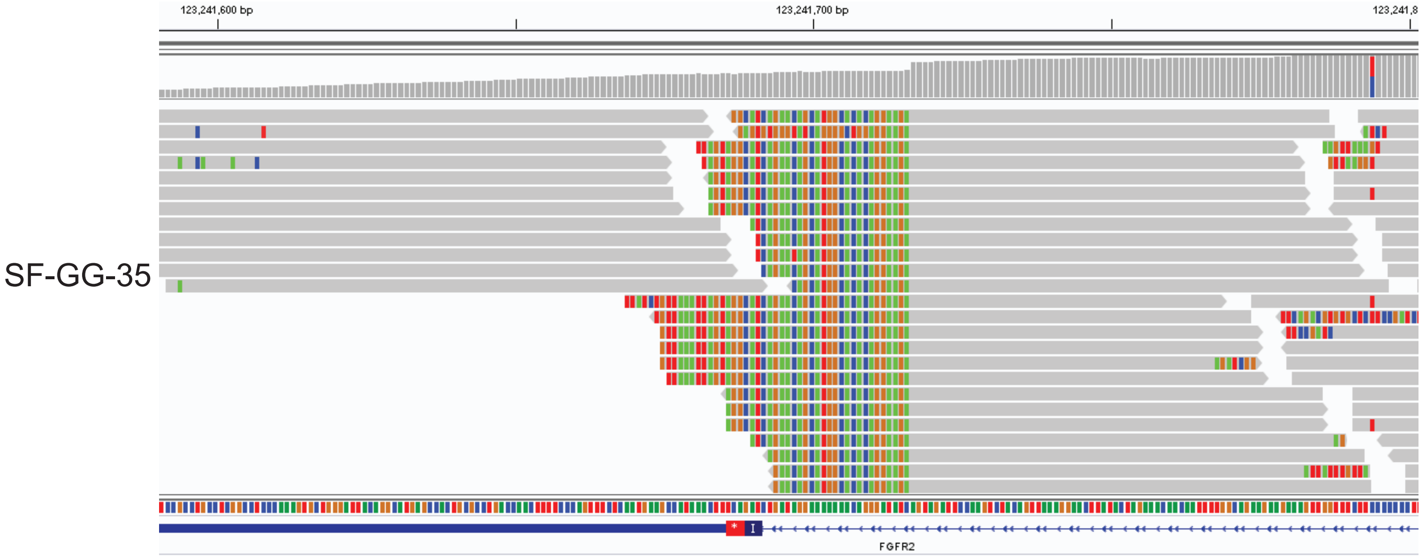


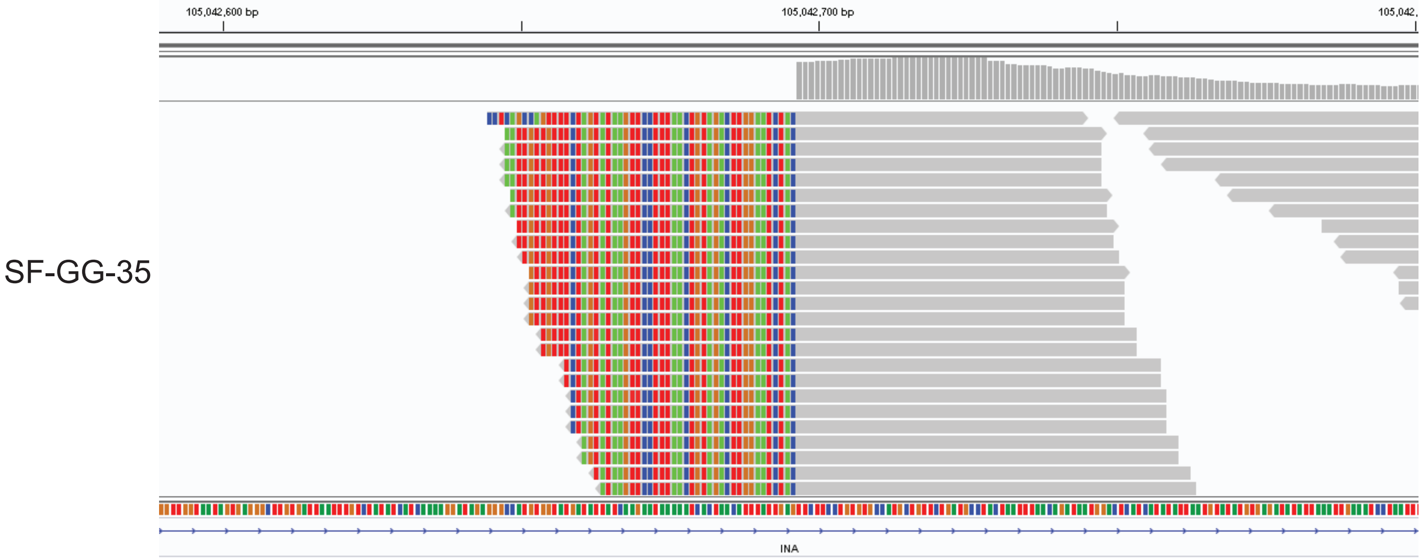

o

*ABL2-GAB2* fusion (exons 1-10 of *ABL2* to exons 5-10 of *GAB2*)

SF-GG-37

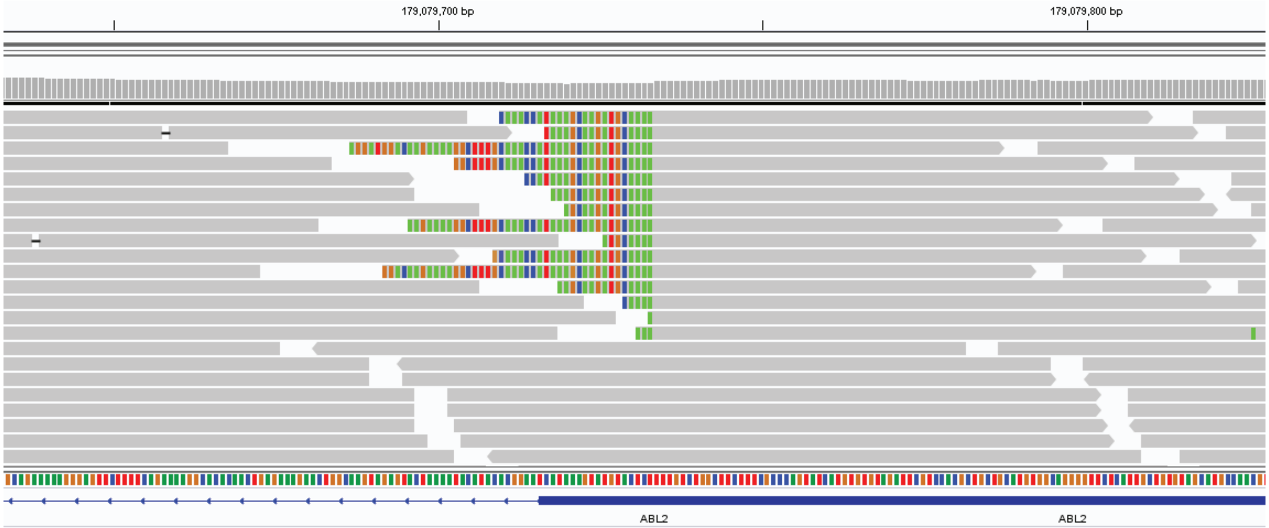


SF-GG-37

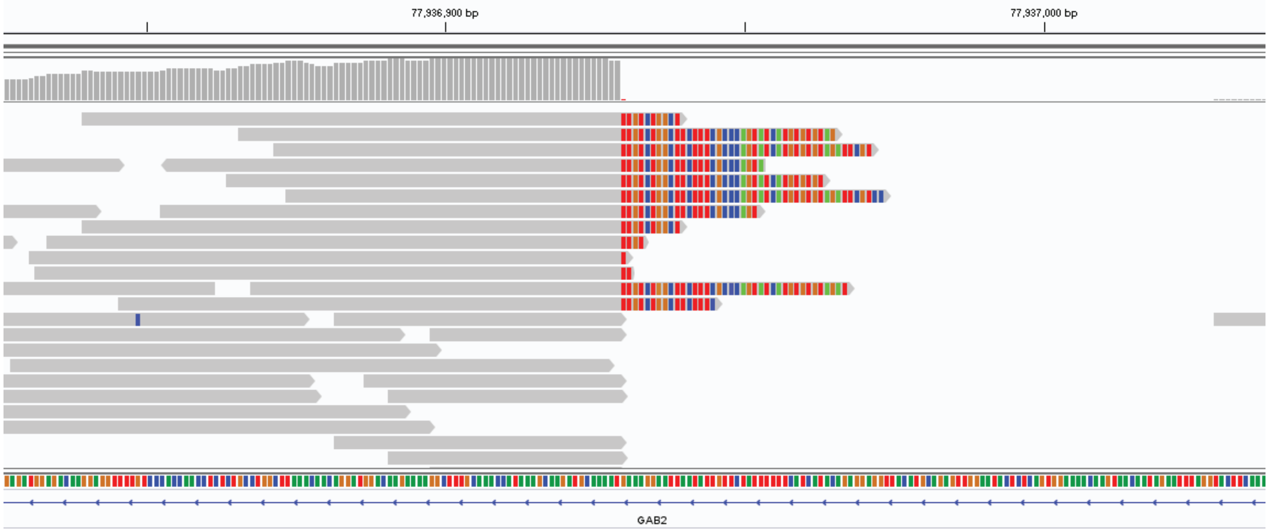

Supplement: Supplementary file 2 — Figures S1-S2. (PDF 7457 kb) [file 40478_2018_551_MOESM2_ESM.pdf]
